# Immunological evaluation of vaccines to prevent *Chlamydia trachomatis* infection using the human in vitro MIMIC system

**DOI:** 10.1093/jimmun/vkag080

**Published:** 2026-04-21

**Authors:** Hong Yu, William M Geisler, Chuanbin Dai, Steffanie Sabbaj, Kanupriya Gupta, Gary Cutter, Guangming Zhong, Robert C Brunham

**Affiliations:** British Columbia Centre for Disease Control, University of British Columbia, Vancouver, British Columbia, Canada; Department of Medicine, University of Alabama at Birmingham, Birmingham, AL, United States; British Columbia Centre for Disease Control, University of British Columbia, Vancouver, British Columbia, Canada; Department of Medicine, University of Alabama at Birmingham, Birmingham, AL, United States; Department of Medicine, University of Alabama at Birmingham, Birmingham, AL, United States; Department of Biostatistics, University of Alabama at Birmingham, Birmingham, AL, United States; Department of Microbiology, Immunology and Molecular Genetics, University of Texas Health Science Center at San Antonio, San Antonio, TX, United States; British Columbia Centre for Disease Control, University of British Columbia, Vancouver, British Columbia, Canada

**Keywords:** attenuated *Chlamydia muridarum* vaccine, cellular immune responses, *Chlamydia trachomatis*, modular immune in vitro construct system, outer membrane protein vaccine

## Abstract

The Modular Immune In vitro Construct (MIMIC) system is an established laboratory-based methodology that reproduces in vitro the human in vivo T-cell immune response. In this study, we utilized the MIMIC system to establish a dendritic cell and CD4^+^ T-cell co-culture model that recapitulates T helper cell responses to *Chlamydia trachomatis* infection in humans. We demonstrate that *C. trachomatis* infection induces *C. trachomatis–*specific IFN-γ–producing CD4^+^ T-cell responses, with IFN-γ as the dominant cytokine, accompanied by significant production of TNF-α, IL-13, IL-8, and IL-6. Live *C. trachomatis* induces stronger IFN-γ CD4^+^ T-cell responses than heat-killed bacteria. A *C. trachomatis* outer membrane protein vaccine comprising the major outer membrane protein and polymorphic membrane proteins PmpE, PmpG, and PmpH elicited limited CD4^+^ T-cell responses, while an attenuated *Chlamydia muridarum* whole cell–based vaccine (intrOv) generated robust CD4^+^ T-cell responses against *C. trachomatis* challenge, encompassing multiple genital *C. trachomatis* serovars. The results suggest that intrOv vaccine should advance along the clinical development pathway as a chlamydia vaccine. MIMIC represents a valuable platform for evaluating chlamydia vaccine candidates, and it can reduce time and cost required to advance vaccine candidates to clinical trials.

## Introduction

One of the major hurdles in developing an effective human chlamydia vaccine is the lack of reliable and sensitive techniques to evaluate vaccine candidates in preclinical settings. Animal models do not always replicate human disease nor predict human immunity. The Modular Immune In vitro Construct (MIMIC) system is a laboratory-based methodology that reproduces the human immune response in vitro. It uses circulating mononuclear cells of individual donors to recapitulate each individual’s immune response, which simulates a clinical trial without putting human subjects at risk.^1–3^ The MIMIC system has been successfully used to predict in vivo protective immune responses to pathogens such as the yellow fever virus^4^ and has the additional advantage of evaluating the impact of human major histocompatibility complex (MHC) diversity on vaccine responses. Evaluation of MIMIC against a range of antigens and commercially available vaccines has shown it to be highly specific in detecting both naïve and memory antigen-specific T responses.^5^^,^^6^

Early *Chlamydia* vaccine trials in humans and nonhuman primates using whole inactivated *Chlamydia trachomatis* ultimately failed because of incomplete and short-lived protection, with even worse inflammation postvaccination in infected nonhuman primates.^7^^,^^8^ Similarly, vaccination against *Chlamydia muridarum* in murine models with inactivated elementary bodies (EBs) conferred only limited protection,^9^^,^^10^ whereas immunization with live *C. muridarum* EBs achieved complete protection against both infection and pathology.^10^^,^^11^ To avoid postvaccination acceleration of pathological inflammation caused by whole-cell vaccines, most contemporary *C. trachomatis* vaccine research has focused on subunit vaccines based on individual protective *C. trachomatis* proteins.^12^^,^^13^

Immunity to *Chlamydia* relies primarily on cell-mediated immune (CMI) responses, particularly CD4^+^ Th1-polarized cytokine responses.^14^^,^^15^ *Chlamydia*-specific CD4^+^ T cells producing IFN-γ play a crucial role in clearing the infection.^16–19^ Our previous proteomic studies identified 5 *Chlamydia* outer membrane proteins (OMPs)—major OMP (MOMP) and polymorphic membrane proteins PmpE, PmpF, PmpG, and PmpH—as potential subunit CD4 T-cell vaccine candidates.^20–22^ In a recent study involving human subjects, we found that IFN-γ responses to these antigens were heterogeneous, with PmpE and MOMP most frequently recognized, especially in women who spontaneously cleared infection.^23^ Because MOMP has multiple serovar sequence variants, immunity to MOMP may be serovar restricted. These observations support a multicomponent vaccine strategy to broaden CD4^+^ T-cell epitope coverage across diverse MHC backgrounds and enhance cross-protection against multiple *C. trachomatis* serovars.

A mutant *C. muridarum* (clone CMG28.51.1), also named intrOv, was recently identified as an attenuated whole-cell potential vaccine candidate.^24^ IntrOv is mapped to loss-of-function mutations in hypothetical genes tc0237 and tc0668 in the *C. muridarum* genome, and is no longer able to induce in mice the development of hydrosalpinx, a putative immune-mediated pathology of the reproductive tract also observed in *C. trachomatis–*infected women.^25^ Oral immunization with intrOv in mice induces robust transmucosal immunity that efficiently protects against infection and reproductive tract pathology caused by wild-type *C. muridarum.*^26^

Adoptive transfer of dendritic cells (DCs) isolated from *Chlamydia*-infected animals has been shown to confer strong protection.^27^ Moreover, *Chlamydia* can survive within DCs, and these infected DCs efficiently present chlamydial antigens.^28^ These findings support the concept that *Chlamydia*-infected DCs are capable of immunizing mice and inducing protective immunity, indicating that *Chlamydia* infection of DCs is relevant to systemic immune responses in the whole animal. In this study, we employed the DC/CD4 co-culture MIMIC system to establish an in vitro model for evaluating CD4^+^ T-cell responses to *C. trachomatis* infection in humans. Using this platform, we compared responses induced by live versus heat-killed (HK) *C. trachomatis* and assessed 2 candidate chlamydia vaccines: a *C. trachomatis* OMP subunit vaccine comprising MOMP, PmpE, PmpG, and PmpH, and an attenuated *C. muridarum* whole cell-based vaccine (intrOv). Our data show that the DC/CD4 co-culture MIMIC system effectively models human CD4^+^ T-cell responses to *C. trachomatis* infection and that live attenuated vaccines induce a better response than subunit protein vaccines. Implications of our study findings are that MIMIC can serve as an informative platform for comparative evaluation of *Chlamydia* vaccine candidates in humans and that the *C. muridarum* intrOv vaccine should be further evaluated in nonhuman primate and human vaccine trials.

## Materials and methods

### Chlamydia

*C. trachomatis* serovar D, E, F, and J strains and *C. muridarum* strain Nigg and its mutant variant intrOv (clone CMG28.51.1) were utilized in the current study. All of these *Chlamydia* organisms were propagated in HeLa 229 cells and purified as EBs as described previously.^29^ HK *C. trachomatis* EBs were prepared by heating at 56 °C for 30 min.

### Recombinant proteins

Four recombinant proteins from *C. trachomatis* serovar D—PmpE (CT869_18-520aa_), PmpG (CT871_25-512aa_), PmpH (CT872_24-520aa_), and MOMP (CT681_24-394aa_)—were obtained from Biomatik (Cambridge, ON, Canada) as N-terminally His-tagged proteins. PmpE, PmpG, and PmpH proteins (N-terminal passenger domain without the signal peptide) and MOMP (full-length protein without the signal peptide) were all produced in an *Escherichia coli* expression system. Each protein was received in lyophilized powder form with purity surpassing 95% and endotoxin <0.1 EU/µg.

### Peripheral blood mononuclear cell source and isolation

Peripheral blood mononuclear cells (PBMCs) were obtained either from fresh whole blood provided by women aged 20–27 years who were prospectively enrolled at the University of Alabama at Birmingham (UAB) Sexual Health Research Clinic or from fresh peripheral blood leukopaks from women aged 18–25 years that were purchased from STEMCELL Technologies (Cat. #200-0092). *C. trachomatis–*seronegative (naïve) and *C. trachomatis–*seropositive subjects were identified by antibody reactivity to *C. trachomatis,* measured by *C. trachomatis* EBs IgG1 and IgG3 ELISA, as previously described.^30^

PBMCs were isolated by Ficoll density gradient centrifugation. Mononuclear cells from the interface were collected, washed twice, and resuspended in serum-free X-VIVO 15 medium (Lonza) for immediate use or cryopreserved in CryoStor CS10 medium (STEMCELL Technologies) and stored in liquid nitrogen for subsequent use.

In total, 23 *C. trachomatis*–naïve subjects (14 recruited from STEMCELL Technologies and 9 from UAB) and 6 *C. trachomatis*–seropositive subjects (from STEMCELL Technologies) were included in this study. PBMCs from these subjects were utilized in different MIMIC assays, depending on experimental design and cell availability. The number of subjects included in each assay is indicated in the corresponding figures.

### Preparation of human DCs

Freshly isolated or cryopreserved PBMCs were used as the source of DCs. Monocytes were purified from total PBMCs using anti-CD14–conjugated magnetic beads (Miltenyi Biotec) and cultured at 7 × 10^5^ cells/mL in a DC medium (serum-free X-VIVO 15 medium [Lonza] supplemented with 100 ng/mL GM-CSF and 25 ng/mL IL-4) for 7 days. On day 5, DCs were pulsed for 24 h with one of the following *Chlamydia* antigens or vaccines: *C. trachomatis* EB (multiplicity of infection [MOI] = 1), *C. muridarum* intrOv EB (MOI = 3), or a *C. trachomatis* OMP combination at a total concentration of 160 ng/mL (40 ng/mL each of MOMP, PmpE, PmpG, and PmpH). The doses of *C. trachomatis* EB, *C. muridarum*, and the *C. trachomatis* OMP combination used for DC pulsing were optimized, as shown in Figs. S2, S4, and S7, respectively. Nonpulsed DCs (no antigen [no Ag] control) were included as a negative control. On day 6, DCs were matured by overnight treatment with 25 ng/mL TNF-α. On day 7, DCs were either co-cultured with naïve CD4^+^ T cells (day 0 of the MIMIC system) to generate antigen-specific CD4^+^ T-cell responses, or co-cultured with antigen-primed CD4^+^ T cells (day 14 of the MIMIC system) to evaluate CD4 T-cell responses against *C. trachomatis*. In this study, *C. trachomatis* in the MIMIC assays refers to serovar D unless otherwise specified (serovars E, F, and J are indicated when used).

### DC/CD4 T-cell co-culture (MIMIC system)

Frozen stocks of autologous PBMCs were used as the source of CD4^+^ T cells. Autologous “untouched” CD4^+^ T cells were purified by negative selection with magnetic beads (Miltenyi Biotec). Purified CD4^+^ T cells (1.5 × 10^6^/well in 1 mL serum-free X-VIVO 15 medium) were plated in 48-well flat-bottom plates. Antigen-pulsed or nonpulsed DCs (2.5 × 10^4^/well in 200 μL serum-free X-VIVO 15 medium), prepared as described above, were added according to experimental design. Co-cultures were maintained in a total volume of 1.2 mL at a CD4^+^ T cell:DC ratio of 60:1 at 37 °C with 5% CO_2_ for 14 days. Culture supernatants were then collected for cytokine analysis using a Meso Scale Discovery (MSD) assay.

**Figure 1.**
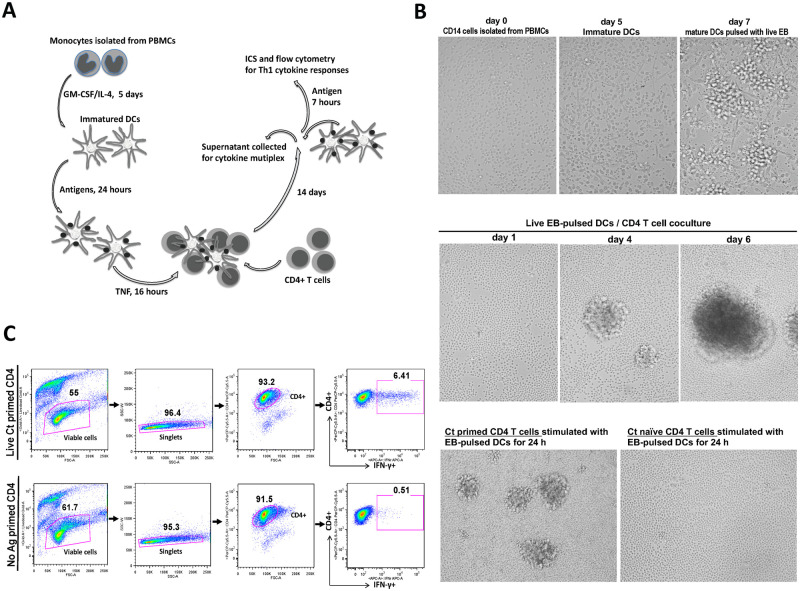
The Modular Immune In vitro Construct (MIMIC) system to measure *Chlamydia trachomatis* (Ct) cellular immune responses in humans. (A) Schematic representation of the MIMIC system workflow. Monocyte-derived DCs were pulsed with antigen and co-cultured with autologous CD4^+^ T cells for 14 days. Supernatants were then collected for cytokine multiplex analysis, and lymphocytes were harvested, restimulated with freshly antigen-pulsed DCs for 7 h, and assessed for Th1 cytokine responses by intracellular cytokine staining (ICS) and flow cytometry. (B) Representative cell images from the MIMIC assay. Top row: monocyte-derived DCs; middle row: live EB-pulsed DC/CD4 T-cell co-culture; bottom row: CD4 memory responses to Ct in the MIMIC system. (C) Gating strategy used to identify IFN-γ–producing CD4^+^ T cells in the MIMIC assay. Flow cytometry analysis illustrates IFN-γ–producing CD4^+^ T cells specific to Ct, induced by a 14-day DC/CD4^+^ T-cell co-culture in a representative Ct-naïve subject.

Antigen-primed CD4^+^ T cells (day 14 of the MIMIC system) were harvested from DC/CD4^+^ T-cell co-cultures and restimulated for 7 h at 37 °C with 5% CO_2_ with autologous DCs pulsed with *C. trachomatis* or nonpulsed (no Ag control) in a 96-well U-bottom plate at a CD4^+^ T cell:DC ratio of 10:1. Brefeldin A (1 μg/mL) was added during the last 5 h of incubation. IFN-γ responses to *C. trachomatis* challenge were assessed by intracellular cytokine staining (ICS) followed by flow cytometry. The workflow of the DC/CD4^+^ T-cell co-culture assay (MIMIC system) is illustrated in Fig. 1A.

To evaluate memory CD4 T-cell responses to *C. trachomatis* infection, a subset of *C. trachomatis–*primed CD4 T cells harvested on day 14 were restimulated with *C. trachomatis–*pulsed DCs at a CD4 T cell:DC ratio of 60:1. Culture supernatants were collected on day 6 for cytokine analysis.

### MSD assay

IFN-γ, TNF-α, IL-1β, IL-2, IL-4, IL-6, IL-8, IL-10, IL-12p70, and IL-13 in the co-culture supernatants were quantified using MSD kit K15049D and a MESO QuickPlex SQ 120MM instrument (Meso Scale Diagnostics, Maryland, USA). The assays were performed according to the manufacturer’s instructions. Results for the MSD assays were reported in pg/mL derived from back-fitting the measured signals for samples to a calibration curve.

### Intracellular staining and flow cytometry

After 7 h of restimulation with autologous DCs pulsed with *C. trachomatis* or nonpulsed (no Ag control), primed CD4^+^ T cells were stained for surface CD4 (PerCP-Cy5.5, clone SK3) and viability using a green fluorescent reactive dye (L23101; Molecular Probes). Cells were then fixed, permeabilized, and stained for intracellular IFN-γ (APC, clone B27) using the BD Cytofix/Cytoperm Plus Fixation/Permeabilization kit according to the manufacturer’s instructions. Flow cytometry data were acquired on a FACSVerse or LSRFortessa system (BD Biosciences) and analyzed with FlowJo software (TreeStar).

### Statistical analysis

Data were analyzed using GraphPad Prism software. Two tailed paired t-tests were used to compare IFN-γ–producing CD4 T cells (by flow cytometry) and cytokine production (by MSD assay) between 2 treatment conditions within the same subject. A 2-tailed ratio paired t-test was applied to compare IFN-γ production in culture supernatants from *C. muridarum* intrOv–, live *C. trachomatis*–, or no Ag–primed CD4 T cells, due to large intersubject differences in scale. *P* values <0.05 were considered statistically significant.

### Study approval

Human peripheral blood was obtained from healthy women with their informed consent and approval from the University of Alabama at Birmingham Institutional Review Board (IRB-300004999). Ethics approval for this study was also obtained from the University of British Columbia Clinical Research Ethics Board (no. H20-01260).

## Results

### The DC/CD4 co-culture MIMIC system was employed as an in vitro human model to recapitulate cellular immune responses to *C. trachomatis* infection

Primary adaptive immune responses are initiated through interactions between rare, naïve antigen-specific T cells and antigen-bearing DCs. The multiple steps for the DC/CD4 co-culture MIMIC system are illustrated in Fig. 1A. We initially used tetanus toxoid (TT) as a positive antigen to establish and validate the DC/CD4 co-culture MIMIC system. As shown in Fig. S1, TT-specific IFN-γ^+^ CD4^+^ responses induced by the MIMIC system were high in a subject vaccinated 3 weeks earlier (14.4%) and much lower in a subject vaccinated 4 years earlier (1.35%). To generate a primary human CD4 T-cell response to *C. trachomatis* infection in vitro, CD14^+^ monocytes isolated from *C. trachomatis*–naïve PBMCs were differentiated into immature DCs by culturing them for 5 days in the DC medium. These immature DCs were then pulsed with live *C. trachomatis* EBs for 24 hours and subsequently matured overnight with TNF-α (Fig. 1B, top row). The dose of *C. trachomatis* EBs for DC pulsing was optimized, and a MOI of 1 was selected for use in the MIMIC system (Fig. S2). Finally, the *C. trachomatis* EB–pulsed DCs were co-cultured with autologous CD4^+^ T cells for 14 days.

During the 14-day co-culture period, we observed the emergence of spotty cell clone growth, which began as tiny clusters and peaked around day 6 (Fig. 1B, middle row). After day 7, however, the majority of activated CD4 T cells underwent cell death, leaving behind only a small fraction that survived as memory cells. On day 14, culture supernatants were harvested for cytokine analysis using the MSD assay, and *C. trachomatis*–primed CD4 T cells were restimulated with *C. trachomatis* EB–pulsed DCs to evaluate *C. trachomatis*–specific IFN-γ production by ICS followed by flow cytometry (Fig. 1A).

As shown in Fig. 1C, the gating strategy and flow cytometry analysis demonstrated *C. trachomatis*–specific IFN-γ–producing CD4 T cells induced by the MIMIC system in a representative *C. trachomatis*–naïve subject. Consistent with this, Fig. 1B (bottom row) illustrates that *C. trachomatis*–primed CD4 T cells from day 14 of the MIMIC system generated robust recall responses when restimulated with EB-pulsed DCs. Notably, within 24 hours of restimulation, large cell clones were visible in the co-culture, whereas no clones were observed when *C. trachomatis*–naïve CD4 T cells were co-cultured with EB-pulsed DCs. These findings indicate that the MIMIC system used in this study recapitulates both primary and memory CD4 T-cell responses to *C. trachomatis* infection.

### The MIMIC system induced *C. trachomatis*–specific cellular immune responses in humans, with IFN-γ as the dominant cytokine, accompanied by significant production of TNF-α, IL-13, IL-8, and IL-6

**Figure 2.**
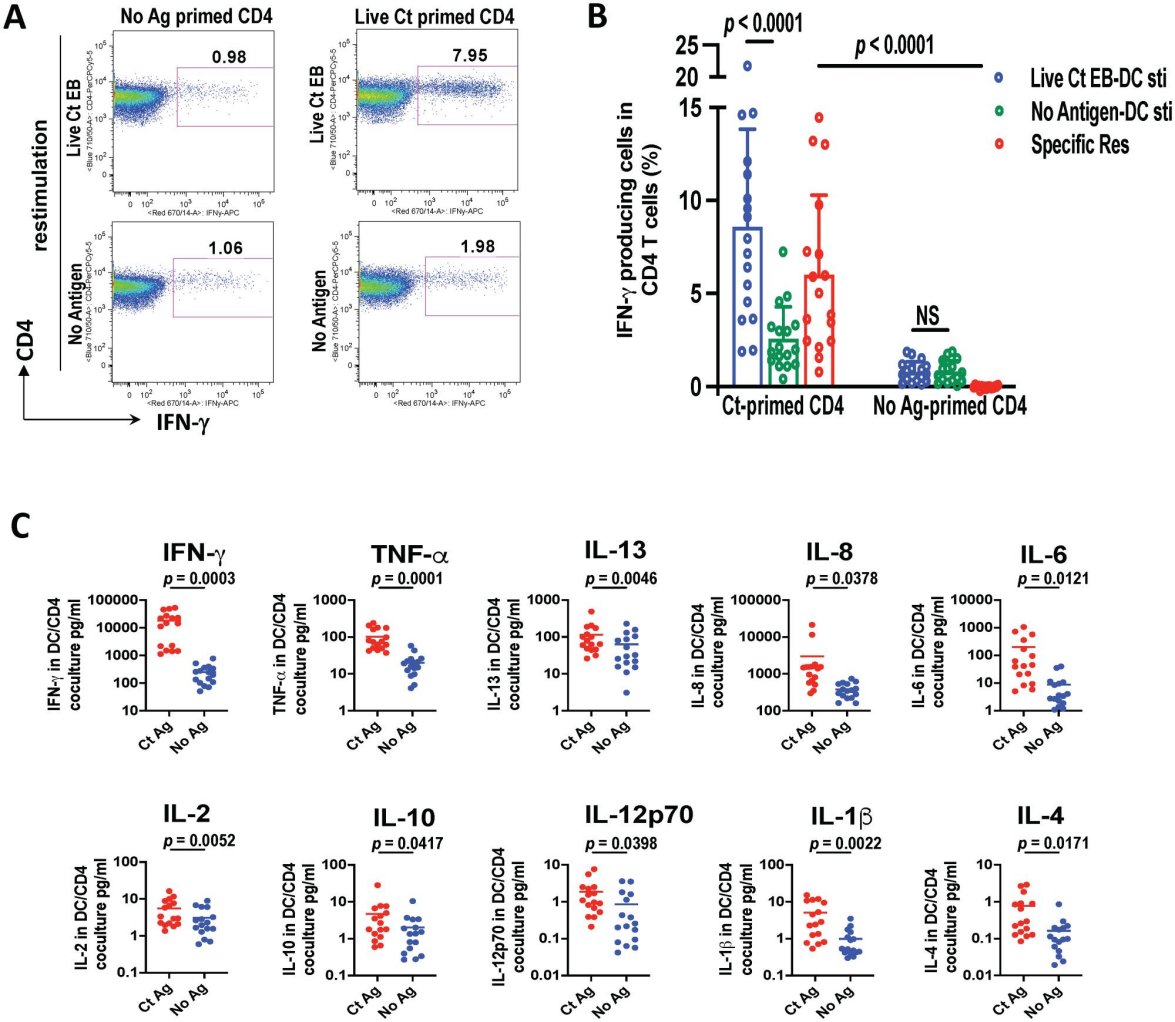
*Chlamydia trachomatis* (Ct)–specific cellular immune responses induced by the MIMIC system in humans. CD4^+^ T cells primed with Ct-primed or unprimed (no Ag) were generated in DC/CD4^+^ T-cell co-cultures by day 14. Culture supernatants were collected to assess cytokine profiles using the MSD assay. Ct-primed and no Ag-primed CD4^+^ T cells were then restimulated with live Ct EB-pulsed DCs or control (no Ag-pulsed) DCs. IFN-γ–producing CD4^+^ T cells were measured by intracellular cytokine staining (ICS) and flow cytometry. (A) Representative flow cytometry plots showing MIMIC-induced IFN-γ–producing CD4^+^ T-cell responses to Ct in a Ct-naïve subject. (B) Frequencies of IFN-γ–producing CD4^+^ T cells to Ct in Ct-naïve subjects (*n* = 16). The percentage of Ct-specific IFN-γ^+^ CD4^+^ T cells was calculated by subtracting the response to no Ag–pulsed DCs from the response to Ct EB–pulsed DCs. (C) Cytokine profiles of CD4^+^ T-cell responses to Ct in Ct-naïve subjects (*n* = 16), determined by MSD assay. Data are mean ± SD in (B) and mean in (C). Two-tailed paired t-tests were used for statistical analysis. A *P* value of <0.05 was considered statistically significant.

Using the established MIMIC system, we evaluated human CD4^+^ T-cell responses to *C. trachomatis* infection in PBMCs from 16 *C. trachomatis*–naïve subjects in vitro. By day 14, live *C. trachomatis*–primed and no Ag–primed CD4^+^ T cells were generated in DC/CD4^+^ T-cell co-cultures and then restimulated with either live *C. trachomatis*–pulsed DCs or control (no Ag–pulsed) DCs. IFN-γ–producing CD4^+^ T cells were quantified by ICS and flow cytometry. Representative flow cytometry plots in DC/CD4^+^ T-cell co-culture from a *C. trachomatis*–naïve subject are shown in Fig. 2A, illustrating MIMIC-induced IFN-γ–producing CD4^+^ T-cell responses to *C. trachomatis*.

As shown in Fig. 2B, C*. trachomatis*–primed CD4^+^ T cells restimulated with live *C. trachomatis* EB–pulsed DCs exhibited significantly higher frequencies of IFN-γ–producing CD4^+^ T cells compared with *C. trachomatis–*primed CD4^+^ T cells restimulated with no Ag–pulsed DCs (*P* < 0.0001). In contrast, no Ag–primed CD4^+^ T cells displayed minimal IFN-γ responses regardless of whether they were restimulated with *C. trachomatis*–pulsed or no Ag–pulsed DCs. When background responses to no Ag–pulsed DCs were subtracted, *C. trachomatis*–primed CD4^+^ T cells showed robust *C. trachomatis*–specific IFN-γ responses that were significantly higher than those of no Ag–primed CD4^+^ T cells (*P* < 0.0001). Notably, no meaningful *C. trachomatis*–specific IFN-γ CD4^+^ T-cell responses were detected in the no Ag–primed group, confirming that the responses observed in *C. trachomatis–*primed CD4^+^ T cells were antigen specific.

Across the 16 subjects, the mean *C. trachomatis*–specific IFN-γ CD4^+^ T-cell response was 5.57 ± 3.98%, ranging from 0.78% to 14.5%. These results demonstrate that *C. trachomatis* infection elicits variable levels of IFN-γ CD4^+^ T-cell responses among different individuals, which may reflect heterogeneity in genetic determinants of host immunity and contribute to differences in clinical outcomes of *C. trachomatis* infection in humans.

To further evaluate CD4^+^ T-cell responses to *C. trachomatis* infection, we quantified 10 cytokines in day 14 co-culture supernatants using the MSD assay, including IFN-γ, TNF-α, IL-1β, IL-2, IL-4, IL-6, IL-8, IL-10, IL-12p70, and IL-13. All 10 cytokines were significantly higher in *C. trachomatis*–primed co-cultures compared with no Ag–primed co-cultures (Fig. 2C). IFN-γ was the predominant cytokine, with a mean concentration of 18,193 ± 17,696 pg/mL in *C. trachomatis*–primed co-cultures versus 253 ± 192 pg/mL in no Ag–primed co-cultures. Among the other cytokines, TNF-α (102 ± 66.5 pg/mL), IL-13 (115 ± 114 pg/mL), IL-8 (2,987 ± 5,548 pg/mL), and IL-6 (201 ± 315 pg/mL) were produced at concentrations >100 pg/mL in *C. trachomatis*–primed co-cultures. By contrast, IL-2 (5.48 ± 4.35 pg/mL), IL-1β (5.09 ± 4.93 pg/mL), IL-10 (4.71 ± 6.72 pg/mL), IL-12p70 (1.88 ± 2.03 pg/mL), and IL-4 (0.77 ± 0.92 pg/mL) were produced at low or very low levels (<10 pg/mL). Thus, the cytokine profile of human *C. trachomatis* infection in the MIMIC system is characterized by IFN-γ as the dominant cytokine, accompanied by substantial production of TNF-α, IL-13, IL-8, and IL-6. Figure S3 shows cytokine dynamics over the 14-day co-culture of cells from a *C. trachomatis–*naïve subject.

### The MIMIC system induced primary and memory CD4^+^ T-cell cytokine responses to *C. trachomatis* in *C. trachomatis–*naïve seronegative subjects, and memory CD4^+^ T-cell cytokine responses in *C. trachomatis–*seropositive subjects

To compare primary and memory human CD4^+^ T-cell responses to *C. trachomatis* infection with this in vitro model, we analyzed cytokine profiles in DC/CD4^+^ T-cell co-culture supernatants collected on day 14 (primary responses) and day 6 after restimulation (memory responses) from 12 *C. trachomatis*–naïve subjects using the MSD assay.

**Figure 3.**
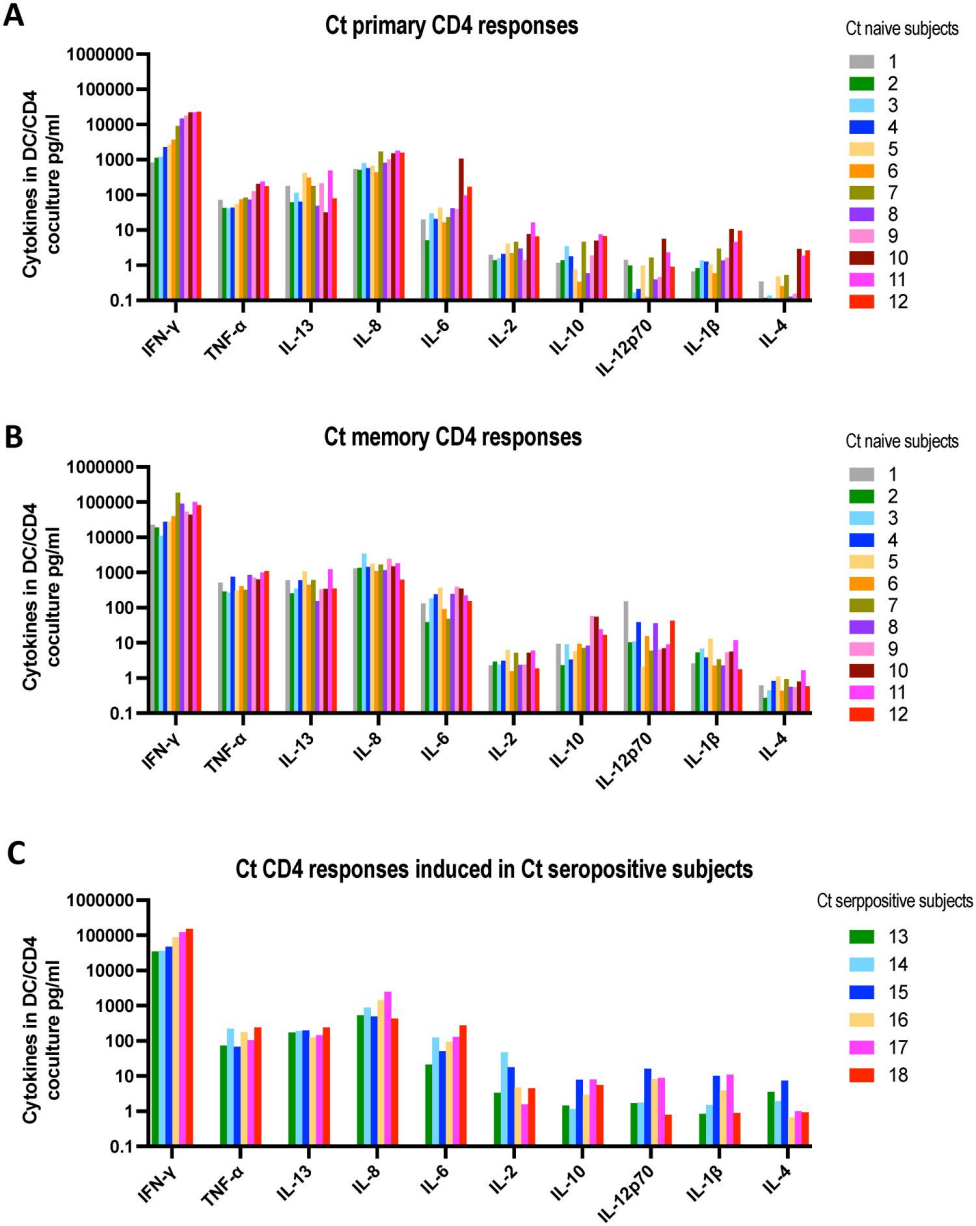
Primary and memory CD4^+^ T-cell cytokine responses to *Chlamydia trachomatis* (Ct) in humans induced by the MIMIC system. (A) Cytokine profiles of primary CD4^+^ T-cell responses to Ct in Ct-naïve subjects. CD4^+^ T cells from 12 Ct-naïve individuals were co-cultured with autologous DCs pulsed with Ct EB for 14 days. Culture supernatants were collected on day 14, and 10 cytokines were measured using the MSD assay. (B) Cytokine profiles of memory CD4^+^ T-cell responses to Ct in Ct-naïve subjects. Ct-primed CD4^+^ T cells (generated via co-culture with Ct EB-pulsed DCs by day 14) from the same 12 Ct-naïve subjects were restimulated with autologous Ct EB–pulsed DCs, respectively. Supernatants were collected on day 6 to assess memory cytokine responses. (C) Cytokine profiles of CD4^+^ T-cell responses to Ct in Ct-seropositive subjects. CD4^+^ T cells from 6 Ct-positive individuals were co-cultured with autologous Ct EB–pulsed DCs for 14 days. Culture supernatants collected on day 14 were analyzed for 10 cytokines using the MSD assay.

Cytokine profiles in both primary (Fig. 3A) and memory (Fig. 3B) responses in the MIMIC system were similar, characterized by IFN-γ as the dominant cytokine, accompanied by substantial production of TNF-α, IL-13, IL-8, and IL-6. However, cytokine levels were significantly higher in memory responses compared to primary responses, including IFN-γ (*P* = 0.0036), TNF-α (*P* < 0.0001), IL-13 (*P* = 0.0002), IL-8 (*P* = 0.0325), IL-12 (*P* = 0.0470), and IL-10 (*P* = 0.0209). Notably, IFN-γ levels increased 10.6-fold in memory responses compared with primary responses. These findings in the MIMIC system reflect the adaptive characteristics of primary versus memory immune responses observed in vivo.

We further extended our investigation to 6 *C. trachomatis*–seropositive subjects identified among the donors and performed the MIMIC assay using the same experimental procedures as in *C. trachomatis*–naïve subjects. In the MIMIC system, the cytokine profile in *C. trachomatis*–seropositive subjects (Fig. 3C) was similar to that observed in *C. trachomatis*–naïve subjects. IFN-γ levels in *C. trachomatis*–seropositive subjects (79,818 ± 489,170 pg/mL) were significantly higher than those observed in primary responses from *C. trachomatis*–naïve subjects (10,062 ± 9,267 pg/mL; *P* = 0.0170) and comparable to memory responses (58,668 ± 49,231 pg/mL). However, other cytokines in *C. trachomatis*–seropositive subjects were not elevated relative to primary responses induced in the MIMIC system. This pattern may reflect differences in immune status: Whereas memory responses induced in the MIMIC system resemble recall responses shortly after a primary infection, cytokine responses in *C. trachomatis*–seropositive subjects may represent more polarized effector T-cell responses that have developed after longer-term in vivo infection.

Overall, these findings demonstrate that in the MIMIC system, both memory responses in *C. trachomatis*–naïve subjects and memory immune responses in seropositive subjects generate significantly higher and comparable levels of IFN-γ production compared with primary responses in naïve subjects. These results suggest that the MIMIC system effectively recapitulates human IFN-γ–mediated immune responses to *C. trachomatis* infection in vivo.

### Live *C. trachomatis* induces stronger CD4^+^ T-cell responses than heat-killed *C. trachomatis* in the MIMIC system, characterized by enhanced IFN-γ production

**Figure 4.**
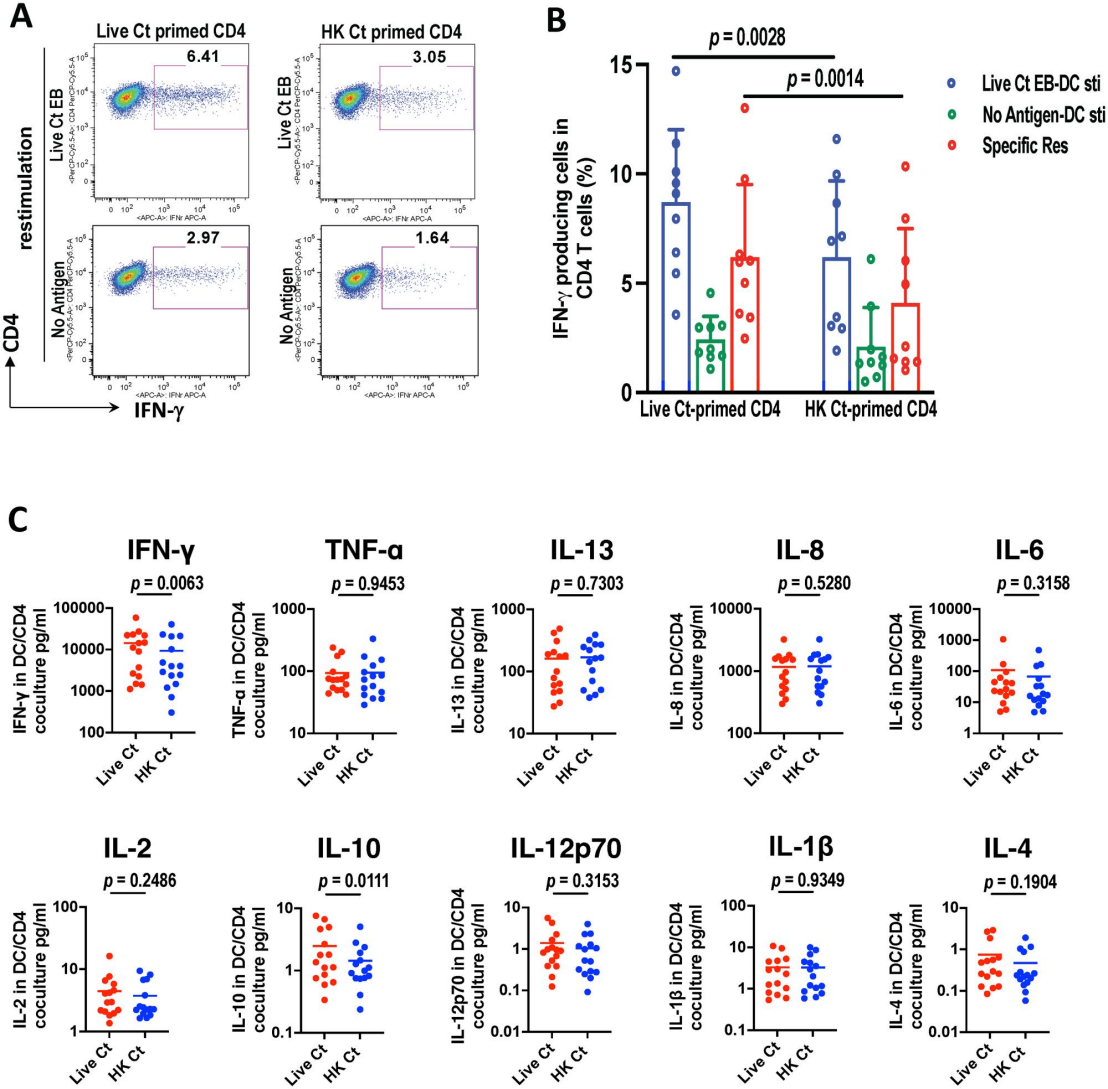
Live vs heat-killed *Chlamydia trachomatis* (Ct)–primed cellular immune responses induced by the MIMIC system in humans. CD4^+^ T cells primed with live Ct or heat-killed (HK) Ct were generated in DC/CD4^+^ T-cell co-cultures by day 14. Culture supernatants were collected to assess cytokine profiles using the MSD assay. Live Ct–primed and HK Ct–primed CD4^+^ T cells were then restimulated with live Ct EB–pulsed DCs or control (no antigen–pulsed) DCs. IFN-γ–producing CD4^+^ T cells were measured by ICS and flow cytometry. (A) Representative flow cytometry plots showing IFN-γ–producing CD4^+^ T-cell responses to Ct induced by live Ct vs HK Ct priming in a Ct-naïve subject. (B) Frequencies of IFN-γ–producing CD4^+^ T cells to *C. trachomatis* in live Ct–primed vs HK Ct–primed CD4^+^ T cells from Ct-naïve subjects (*n* = 9). Ct-specific IFN-γ^+^ CD4^+^ T-cell responses were calculated by subtracting responses to no Ag–pulsed DCs from those to live Ct EB–pulsed DCs. (C) Cytokine profiles of CD4^+^ T-cell responses primed with live Ct vs HK Ct in Ct-naïve subjects (*n* = 15), determined by MSD assay. Data are mean ± SD in (B) and mean in (C). Two-tailed paired t-tests were used for statistical analysis. A *P* value of <0.05 was considered statistically significant.

To investigate potential protective immune mechanisms, we compared CD4^+^ T-cell responses induced by live versus HK *C. trachomatis* in the MIMIC system. By day 14, live *C. trachomatis*–primed and HK *C. trachomatis*–primed CD4^+^ T cells from naïve subjects were generated in DC/CD4^+^ T-cell co-cultures and subsequently restimulated with either live *C. trachomatis*–pulsed DCs or control (no Ag–pulsed) DCs. IFN-γ–producing CD4^+^ T cells were quantified by flow cytometry. Representative plots of IFN-γ–producing CD4^+^ T cells induced by live versus HK *C. trachomatis* are shown in Fig. 4A.

As summarized in Fig. 4B, live *C. trachomatis*–primed CD4^+^ T cells restimulated with *C. trachomatis*–pulsed DCs exhibited significantly higher frequencies of IFN-γ–producing CD4^+^ T cells compared with HK *C. trachomatis*–primed CD4^+^ T cells (*P* = 0.0028). Likewise, after subtracting background responses to no Ag–pulsed DCs, *C. trachomatis–*specific IFN-γ responses were significantly greater in live *C. trachomatis*–primed compared to HK *C. trachomatis*–primed CD4^+^ T cells (*P* = 0.0014).

We further evaluated live versus HK *C. trachomatis*–induced CD4^+^ T-cell responses by quantifying 10 cytokines in day 14 co-culture supernatants (Fig. 4C) in 15 *C. trachomatis–*naïve subjects. IFN-γ production was significantly higher in live *C. trachomatis*–primed compared with HK *C. trachomatis*–primed co-cultures (*P* = 0.0063). No significant differences were observed for the other 9 cytokines, except IL-10 (*P* = 0.0111). However, given that IL-10 levels in all tested samples were very low (<10 pg/mL), the biological relevance of this difference is uncertain.

Together, these findings demonstrate that live *C. trachomatis* preferentially induces stronger IFN-γ–producing CD4^+^ T-cell responses than HK bacteria in the MIMIC system, consistent with IFN-γ–producing CD4^+^ T cells as a key correlate of protective immunity against *C. trachomatis*.

### A *C. trachomatis* OMP vaccine comprising MOMP, PmpE, PmpG, and PmpH elicited limited CD4^+^ T-cell responses against *C. trachomatis* challenge in the MIMIC system

**Figure 5.**
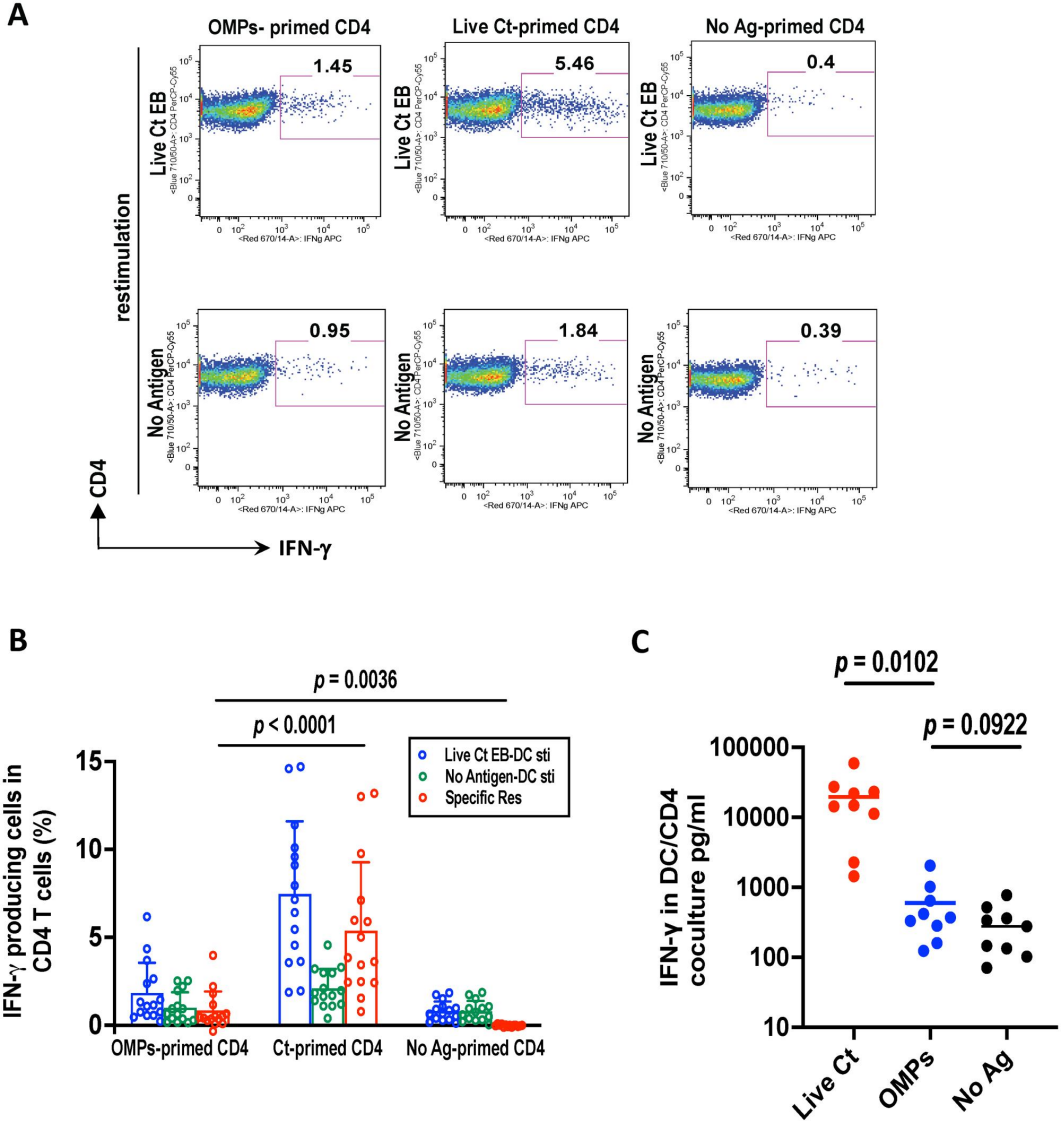
*Chlamydia trachomatis* (Ct) outer membrane protein (OMP) vaccine–induced cellular immune responses evaluated by the MIMIC system in humans. CD4^+^ T cells primed with Ct OMPs, live Ct, or no antigen (no Ag) were generated in DC/CD4^+^ T-cell co-cultures by day 14. These primed T cells were then restimulated with live Ct EB–pulsed DCs or control (no Ag–pulsed) DCs. IFN-γ–producing CD4^+^ T cells were measured by ICS and flow cytometry. Culture supernatants were collected to assess IFN-γ production using the MSD assay. (A) Representative flow cytometry plots showing IFN-γ–producing CD4^+^ T-cell responses to Ct induced by OMP vaccine, live Ct, or no Ag control in a Ct-naïve subject. (B) Frequencies of IFN-γ–producing CD4^+^ T cells to Ct in OMP-, live Ct–, or no Ag–primed CD4^+^ T cells from Ct-naïve subjects (*n* = 15). Ct-specific IFN-γ^+^ CD4^+^ T-cell responses were calculated by subtracting responses to no Ag–pulsed DCs from those to live Ct EB–pulsed DCs. (C) IFN-γ production in culture supernatants from OMP, live Ct– or no Ag–primed CD4^+^ T cells in Ct-naïve subjects (*n* = 9). Data are mean ± SD in (B) and mean in (C). Two-tailed paired t-tests were used for statistical analysis. A *P* value of <0.05 was considered statistically significant.

Using the MIMIC system, we evaluated a vaccine combination of 4 *C. trachomatis* OMPs (MOMP, PmpE, PmpG, and PmpH) for their ability to induce CD4^+^ T-cell responses against *C. trachomatis*. The concentration of OMP antigens for DC pulsing was optimized, and the total protein concentration of 160 ng/mL (40 ng/mL each of PmpE, PmpG, PmpH, and MOMP) was selected for use in the MIMIC system (Fig. S4). By day 14, OMPs primed CD4^+^ T cells generated in DC/CD4^+^ T-cell co-cultures were restimulated with either live *C. trachomatis* EB–pulsed DCs or control (no Ag pulsed) DCs, and IFN-γ–producing CD4^+^ T cells were quantified as a readout of vaccine efficacy. *C. trachomatis*–primed CD4^+^ T cells and no Ag–primed CD4^+^ T cells were included as positive and negative controls, respectively. Representative flow cytometry plots of IFN-γ–producing CD4^+^ T cells induced by the OMP vaccine, live *C. trachomatis*, and no Ag control are shown in Fig. 5A.

As summarized in Fig. 5B, among 15 *C. trachomatis*–naïve subjects, the frequencies of *C. trachomatis*–specific IFN-γ–producing CD4^+^ T cells induced by the OMP vaccine, live *C. trachomatis*, and no Ag control were 0.835 ± 1.09%, 5.37 ± 3.9%, and −0.0154 ± 0.0596%, respectively. Although OMP vaccine–primed CD4^+^ T cells showed significantly higher frequencies of *C. trachomatis*–specific IFN-γ–producing CD4^+^ T cells compared with the no Ag control (*P* = 0.0036), they were markedly lower than those induced by live *C. trachomatis* (*P* < 0.0001). Consistent with this, IFN-γ levels in co-culture supernatants were significantly lower for the OMP vaccine compared with live *C. trachomatis* (*P* = 0.0102) and did not differ from the no Ag control (*P* = 0.0922) (Fig. 5C). A MIMIC assay with the 4 individual OMP vaccines showed that the OMP combination produced an additive effect, with MOMP as the major contributor to immune responses, followed by PmpE, PmpG, and PmpH (Fig. S5).

Together, these findings indicate that the *C. trachomatis* OMP vaccine combination of MOMP, PmpE, PmpG, and PmpH elicited limited CD4^+^ T-cell responses against *C. trachomatis* challenge in the MIMIC system.

### IntrOv, an attenuated *C. muridarum* vaccine, elicits robust human CD4^+^ T-cell responses with high IFN-γ production across multiple genital *C. trachomatis* serovar challenges in the MIMIC system

Because the *C. trachomatis* OMP vaccine combination induced limited immunological responses in the MIMIC system and primary *C. muridarum* protects against *C. trachomatis* challenge in the murine system,^31^ we were interested in evaluating the attenuated *C. muridarum* vaccine (intrOv) in the MIMIC system. Thus, we investigated the presence of cross-protective immune responses between *C. muridarum* and *C. trachomatis* in a human setting. We utilized the MIMIC system to generate CD4 T cells primed with *C. muridarum* or *C. trachomatis* serovar D, along with no Ag–primed CD4 cells as a negative control, from 2 *C. trachomatis*–naïve subjects, respectively. Our results revealed that *C. muridarum–*primed CD4 T cells from both subjects exhibited potent IFN-γ responses when challenged with *C. trachomatis*. Interestingly, the IFN-γ CD4 T-cell responses induced by *C. muridarum* were even more robust compared to that induced by *C. trachomatis* (Fig. S6). The dose of *C. muridarum* for DC pulsing was optimized, and a MOI of 3 was selected for use in the MIMIC system (Fig. S7).

**Figure 6.**
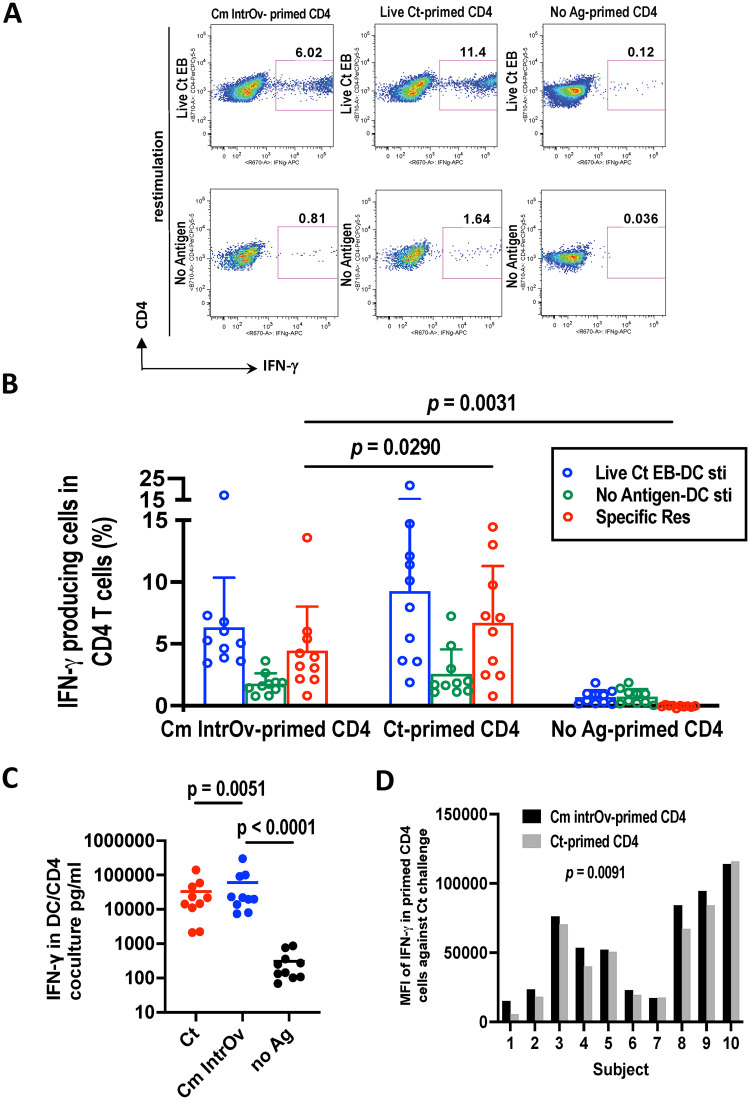
*Chlamydia muridarum*–based vaccine (Cm intrOv)–induced cellular immune responses evaluated by the MIMIC system in humans. CD4^+^ T cells primed with Cm intrOv, live *C. trachomatis* (Ct), or no antigen (no Ag) were generated in DC/CD4^+^ T-cell co-cultures by day 14. Culture supernatants were collected to assess IFN-γ production using the MSD assay. These primed CD4^+^ T cells were then restimulated with live Ct EB–pulsed DCs or control (no Ag–pulsed) DCs. IFN-γ–producing CD4^+^ T cells were measured by ICS and flow cytometry. (A) Representative flow cytometry plots showing IFN-γ–producing CD4^+^ T-cell responses to Ct induced by Cm intrOv, live Ct, or no Ag control in a Ct-naïve subject. (B) Frequencies of IFN-γ–producing CD4^+^ T cells to Ct in Cm intrOv-, live Ct–, or no Ag–primed CD4^+^ T cells from Ct-naïve subjects (*n* = 10). Ct-specific IFN-γ^+^ CD4^+^ T-cell responses were calculated by subtracting responses to no Ag–pulsed DCs from those to live Ct EB–pulsed DCs. (C) IFN-γ production in culture supernatants from Cm intrOv–, live Ct–, or no Ag–primed CD4^+^ T cells in Ct-naïve subjects (*n* = 10). (D) Mean fluorescence intensity (MFI) of IFN-γ in Cm intrOv–primed vs live Ct–primed CD4^+^ T cells in response to Ct challenge. Data are mean ± SD in (B) and mean in (C). Two-tailed paired t-tests in (B) and (D) and ratio paired t-tests in (C) were used for statistical analysis. A *P* value of <0.05 was considered statistically significant.

To evaluate the intrOv vaccine in the MIMIC system, CD4^+^ T cells from 10 *C. trachomatis*–naïve subjects were primed using the MIMIC system and restimulated with either live *C. trachomatis*–pulsed DCs or control (no Ag–pulsed) DCs. Representative flow cytometry plots of IFN-γ–producing CD4^+^ T cells induced by the intrOv vaccine, live *C. trachomatis*, and no Ag control are shown in Fig. 6A.

As summarized in Fig. 6B, intrOv-primed CD4^+^ T cells from *C. trachomatis*–naïve subjects restimulated with live *C. trachomatis*–pulsed DCs showed significantly higher frequencies of *C. trachomatis*–specific IFN-γ–producing CD4^+^ T cells compared with no Ag–primed controls (4.45 ± 3.57% vs −0.044 ± 0.085%; *P* = 0.0031). This was slightly lower than live *C. trachomatis–*primed cells (6.69 ± 4.61%; *P* = .0290). Of note, IFN-γ levels in intrOv co-culture supernatants were higher than in live *C. trachomatis* co-cultures (60,535 ± 90,462 pg/mL vs 33,443 ± 41,731 pg/mL; *P* = 0.0051) (Fig. 6C), and the mean fluorescence intensity of IFN-γ in intrOv-primed CD4^+^ T cells responding to *C. trachomatis* challenge was higher than in live *C. trachomatis*–primed cells in 8 of 10 subjects tested (*P* = 0.0091) (Fig. 6D), indicating strong functional activation per responding cell.

**Figure 7.**
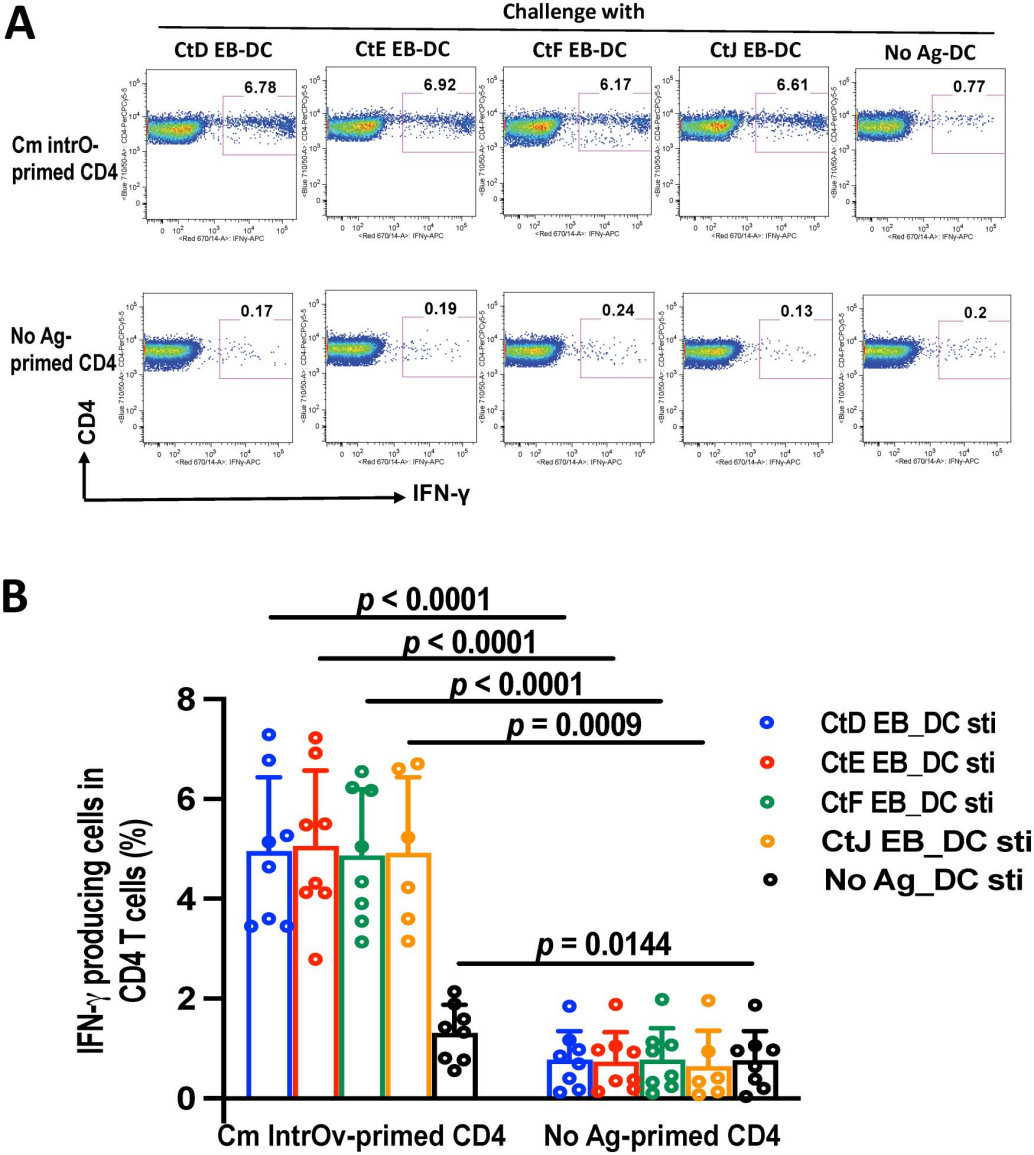
*Chlamydia muridarum*–based vaccine (Cm intrOv)–induced CD4^+^ T-cell responses against multiple genital *C. trachomatis* (Ct) serovar challenges evaluated by the MIMIC system in humans. CD4^+^ T cells primed with Cm intrOv or no antigen (no Ag) were generated in DC/CD4^+^ T-cell co-cultures by day 14. Cm intrOv–primed or no Ag–primed CD4^+^ T cells were then challenged with DCs pulsed with Ct serovar D, E, F, or J, or with control (no Ag–pulsed) DCs. (A) Representative flow cytometry plots showing IFN-γ–producing CD4^+^ T-cell responses against multiple genital Ct serovars in a Ct-naïve subject. (B) Frequencies of IFN-γ–producing CD4^+^ T cells against multiple genital Ct serovars in Cm intrOv–primed vs no Ag–primed CD4^+^ T cells from Ct-naïve subjects (*n* = 8). Data are mean ± SD. Two-tailed paired t-tests were used for statistical analysis. A *P* value of <0.05 was considered statistically significant.

To assess the breadth of immunity in the MIMIC system, intrOv-primed CD4^+^ T cells were challenged with live *C. trachomatis* EB–pulsed DCs representing serovars D, E, F, and J, or control DCs. Representative flow cytometry plots of IFN-γ–producing CD4^+^ T cells against multiple genital serovar challenges induced by the intrOv vaccine and no Ag control are shown in Fig. 7A.

As shown in Fig. 7B, intrOv-primed CD4^+^ T cells from 8 *C. trachomatis*–naïve subjects elicited robust *C. trachomatis*–specific IFN-γ responses against all 4 serovars: D (4.95 ± 1.48%), E (5.06 ± 1.51%), F (4.87 ± 1.33%), and J (4.92 ± 1.52%). These responses were significantly higher than those of the no Ag–primed controls (*P* < 0.0001 for challenge with serovars D, E, and F; *P* = 0.0009 for challenge with serovar J). We also observed slightly higher background IFN-γ responses in the intrOv-primed group than the no Ag–primed group when challenged with no Ag–pulsed DCs (*P* = 0.0144). However, after subtracting background responses, intrOv-primed CD4^+^ T cells continued to show strong and significantly elevated *C. trachomatis*–specific IFN-γ responses compared with no Ag–primed CD4^+^ T cells (*P* = 0.0003 for serovar D; *P* = 0.0002 for serovars E and F; *P* = 0.0012 for serovar J), confirming the specificity of the vaccine-induced responses.

Taken together, these results demonstrate that the intrOv vaccine in the MIMIC system elicits robust human CD4^+^ T-cell responses with high IFN-γ production and broad immune reactivity to multiple genital *C. trachomatis* serovars, supporting its further clinical development as a vaccine to prevent human *C. trachomatis* infection and disease.

## Discussion

To date, deliberately infecting humans with *Chlamydia trachomatis* has not been deemed safe or practical, leading to the absence of human challenge studies. Withholding treatment for known infections is considered ethically unacceptable, which hampers direct observation of the natural course of *Chlamydia* infection in humans. In this study, we present data from an innovative method, the in vitro MIMIC system, which replicates in vitro the human cellular immune responses to *C. trachomatis* infection. Using this system, we successfully measured the primary and memory CD4 T-cell responses in *Chlamydia-*naïve subjects as well as *C. trachomatis* responses in the *C. trachomatis*–seropositive subjects. Our results shed light on the cytokine responses in *C. trachomatis* infection, with IFN-γ being the dominant player, alongside significant contributions from TNF-α, IL-13, IL-8, and IL-6.

IFN-γ remains the central mediator of protection against *C. trachomatis*, enhancing antimicrobial activity in epithelial cells and macrophages and restricting intracellular bacterial replication.^12^^,^^32^ TNF-α acts synergistically with IFN-γ to limit chlamydial growth, while also amplifying inflammatory cell recruitment and possibly contributing to genital tract tissue damage.^33^ IL-8 functions as a potent neutrophil chemoattractant that facilitates early clearance, consistent with our observation of elevated IL-8 as early as 1 day postinfection in the MIMIC system (Fig. S3). While *Chlamydia-*infected epithelial cells are known to release IL-8 as part of the innate immune response,^34^ our data indicate that DC/CD4^+^ co-cultures represent an additional significant source of IL-8, likely derived primarily from DCs, in response to *Chlamydia* infection. However, sustained IL-8 production may also drive collateral tissue injury.^33^^,^^35^ IL-6 further promotes local inflammation and supports Th17 differentiation,^36^ which may enhance mucosal defense but may also perpetuate inflammatory pathology. IL-13 is traditionally considered a Th2-associated cytokine implicated in fibrosis and tissue remodeling.^37^ Interestingly in the murine system, a novel genital tract-resident CD4^+^ T-cell subset (CD4γ13 T cells) has been described that co-produces IFN-γ and IL-13. Adoptive transfer of a *Chlamydia*-specific CD4γ13 T-cell clone completely prevented oviduct immunopathology without accelerating bacterial clearance.^38^ In humans, a PBMC IL-13 response to *C. trachomatis* EBs has been associated with resistance to reinfection.^19^ These findings suggest that IL-13, or IL-13–producing cells, may exert divergent roles in *Chlamydia* infection, contributing both to protective mechanisms and to immunopathology.

Although IFN-γ is the dominant cytokine detected across all subjects in our in vitro *C. trachomatis* infection model, we did observe substantial variations in cytokine production between individuals. For instance, among the 12 subjects tested (Fig. 3), IFN-γ levels varied by up to 28-fold in primary responses and up to 17-fold in memory responses. Similarly, IL-6 production differed by up to 211-fold in primary responses and up to 10-fold in memory responses. Such variability in cytokine magnitude and in their dual roles in protection and pathology may underlie divergent outcomes of chlamydial infection in individuals. A promising application of the MIMIC *C. trachomatis* infection model will be to generate CD4/DC–*C. trachomatis* co-cultures from patients with distinct clinical outcomes and apply high-throughput tools such as microarrays to identify immunologic biomarkers and predictors of persistence, pathogenesis, and protective immunity.

Whole inactivated *C. trachomatis* vaccines in humans provided only transient protection and may even have worsened inflammation. Live *C. muridarum* in mice confers complete protection against both infection and pathology, while inactivated *C. muridarum* exhibits only limited protection. We also previously demonstrated that dead *C. muridarum* EBs loaded fewer peptides onto MHC class II (MHC-II) molecules than live *C. muridarum* infection of murine DCs.^39^ These findings highlight the importance of defining immune correlates that distinguish protective from nonprotective responses following immunization with dead versus live organism. Thus, it would be particularly informative to compare CD4^+^ T-cell responses to live versus inactivated *C. trachomatis* using the MIMIC system. Our data show that live *C. trachomatis* induces significantly stronger CD4^+^ T-cell responses than HK bacteria, characterized by higher frequencies of IFN-γ–producing CD4^+^ T cells and elevated IFN-γ secretion. Other cytokines were largely unchanged, indicating that live organisms preferentially promote IFN-γ–driven CD4^+^ T-cell immunity and reinforcing IFN-γ as a central correlate of protection and underscoring its role in vaccine immunity.

Although our prior human studies did not identify IFN-γ production in response to EBs as a correlate of protection,^19^^,^^40^ these investigations demonstrated that certain *C. trachomatis* antigen-specific immune responses can alter susceptibility to reinfection. Bakshi et al. reported that women with *C. trachomatis*–specific IFN-γ–producing CD4^+^ T-cell responses at follow-up were significantly less likely to experience reinfection; responses to MOMP accounted for protection in approximately 40% of resistant individuals, implying that immunity in the remaining subjects was mediated by responses to other *C. trachomatis* antigens.^40^ In a separate cohort, Cohen et al. observed that antigen-specific IFN-γ production in response to heat-shock protein 60 was correlated with a reduced incidence of new infection among high-risk Kenyan women.^19^ Given that *Chlamydia* encodes approximately 900 proteins, these observations suggest that protective immunity is mediated by responses to only a limited subset of T-cell antigens.

The development of an in vitro assay to prime human responses is of significant value for the preclinical evaluation of vaccine candidates. Gaucher et al. conducted a study using an in vitro DC/CD4 co-culture MIMIC system to define the signature of the immune response to the yellow fever vaccine 17D (YF17D) in humans. The study demonstrated that the in vitro co-culture MIMIC system primed and challenged with YF17D virus elicited robust and diverse primary T helper cell responses in naïve individuals. Of central importance, microarray analysis confirmed that the same molecular factors required for mounting a protective response were activated by both in vivo vaccination with YF17D and in vitro co-culture system of primary immune response to the virus.^4^

In this study, we applied the MIMIC system to evaluate a *C. trachomatis* OMP vaccine combination comprising MOMP, PmpE, PmpG, and PmpH in humans. Our earlier work demonstrated that *Chlamydia* OMPs preferentially provide MHC-II peptides, and that a recombinant vaccine containing MOMP and PmpE, PmpF, PmpG, and PmpH with a Th1-polarizing adjuvant significantly accelerated clearance in murine infection models.^22^ These findings supported *Chlamydia* OMPs as relevant T-cell antigens for subunit vaccine development. However, in the MIMIC system, the OMP combination elicited only modest and variable CD4^+^ T-cell responses following *C. trachomatis* challenge. Direct comparison of a viable intracellular bacterium that infects and activates human dendritic cells with a limited set of recombinant exogenous proteins represents an inherently unequal comparison. In addition, the OMP antigens were His-tagged recombinant fusion proteins; complexing them with anti–His tag antibodies could facilitate Fc receptor-dependent antigen presentation and enhance CD4^+^ T-cell activation. The modest responses observed may also reflect a low precursor frequency of cognate T cells in humans, consistent with the prolonged course of infection resolution in humans (months to years) compared with mice (5 to 6 weeks). Future studies incorporating immune complex-mediated delivery or optimized formulation strategies, including appropriate adjuvants, may improve immunogenicity and increase the predictive value of the MIMIC system for subunit vaccine development.

The extraordinary similarity in gene content and order between *C. muridarum* and *C. trachomatis*^41^ opens up exciting prospects for developing a heterologous cross-protective vaccine. Ramsey et al. previously described the cross-species or heterotypic protective immunity in a mouse model of *Chlamydia* genital tract infection.^31^ In their study, mice primed by genital infection with *C. muridarum* were protected against subsequent challenge with either of 2 human *C. trachomatis* serovars, E or L2. Analyses of blast transformation and delayed-type hypersensitivity responses^31^ demonstrated that *C. muridarum* infection induced broadly cross-reactive T cells capable of recognizing epitopes from human *C. trachomatis* biovars. Immunoblotting showed that *C. muridarum* infection elicited IgG against human serovar E MOMP as well as other distinct antigens, suggesting a role for antibodies in protection, including neutralization and Fc receptor–mediated antigen presentation. The authors further proposed that, under conditions of high challenge doses, the observed cross-protective immunity was primarily T-cell mediated. This interpretation is supported by the observation that most antibody epitopes identified localize to variable regions of MOMP that differ among serovars or serogroups,^42^ whereas T-cell epitopes are frequently located within highly conserved regions.^43^^,^^44^ Kaltenboeck et al. identified candidate T-cell epitopes within MOMP that are shared between human and murine biovars.^43^ Ortiz and colleagues identified a single immunodominant MOMP region containing up to 6 potential epitopes capable of eliciting HLA-restricted responses in the majority of infected women.^44^ Notably, this sequence is conserved in the *C muridarum* MOMP.^31^ Collectively, these findings suggest that multiple broadly cross-reactive T-cell epitopes within MOMP may represent an important mechanism underlying *C. muridarum*–induced protection against *C. trachomatis*.

IntrOv, a mutant clone of *C. muridarum*, has been developed as a genital pathogenicity-attenuated whole cell vaccine to prevent human *C. trachomatis* infection and disease. Using the MIMIC system, we demonstrated that the intrOv vaccine elicits robust human IFN-γ–producing CD4^+^ T-cell responses against multiple genital *C. trachomatis* serovars, thereby overcoming the potential limitation of serovar-specific immunity. Notably, IFN-γ production per cell was higher in intrOv primed co-cultures than in *C. trachomatis*–primed co-cultures. This difference may reflect the optimized dosing used for DC pulsing (MOI 3 for *C. muridarum* vs MOI 1 for *C. trachomatis*), subtle sequence differences in immunodominant antigens between the 2 species, or the absence of immunosuppressive mechanisms that *C. trachomatis* may have evolved during its long association with humans. Although the MIMIC platform has been successfully applied to viral vaccines such as influenza and yellow fever, pathogens with relatively limited proteomes, *Chlamydia*, which encodes approximately 900 proteins, requires a more cautious interpretation of MIMIC-derived data. Accordingly, while the attenuated *C. muridarum* intrOv strain exhibited robust immunogenicity in this in vitro system, these responses should not be assumed to translate directly into in vivo protection and will require validation in appropriate animal models. Collectively, our findings support intrOv as a promising attenuated *Chlamydia* vaccine candidate for future preclinical and clinical evaluation.

It should be noted that the total frequency of IFN-γ–producing CD4^+^ T cells may not be strictly predictive of vaccine-elicited protection. Rather, multifunctional CD4^+^ T cells coexpressing IFN-γ and TNF-α have been shown to correlate more closely with protective immunity than IFN-γ single-positive cells in murine models of *C. muridarum* infection,^39^ as well as in other pathogens such as *Leishmania major*^45^ and *Mycobacterium tuberculosis.*^46^ Accordingly, in the present study we assessed IFN-γ/TNF-α double-positive cells within the total IFN-γ–producing CD4^+^ T-cell population induced under different conditions in the MIMIC system. The majority of IFN-γ–producing CD4^+^ T cells generated in the live *C. trachomatis*, HK *C. trachomatis*, OMP, or *C. muridarum* intrOv conditions coexpressed TNF-α. Notably, the proportion of IFN-γ/TNF-α double-positive cells induced by *C. muridarum* intrOv was significantly higher than that induced by the OMP vaccine, suggesting that intrOv may promote a more multifunctional CD4^+^ T-cell response associated with protective immunity (Fig. S8).

Based on our own and others’ experience with the MIMIC system, several technical considerations are essential to ensure reliable induction of naïve and memory human CD4^+^ T-cell responses. Serum-free media such as X-VIVO 15 and CryoStor CS10 minimize nonspecific antigen exposure and reduce background responses. Although PBMCs are ideally processed immediately after blood or leukopak collection, in this study they were processed within 24 h of collection (cell viability >99%) due to shipping from the United States to Canada. Longer shipping times, even with viability >97%, led to markedly elevated background responses or even DC preparation failure. Gentle handling of DCs is also essential to preserve cell integrity and prevent nonspecific activation. Finally, optimizing CD4^+^ T-cell and DC ratios is crucial for effective priming, and appropriate negative controls are necessary to confirm antigen-specific responses. Together, these factors establish the conditions under which the MIMIC assay can effectively model human CD4^+^ T-cell priming.

In conclusion, the DC/CD4 co-culture MIMIC system provides a novel model of human T helper cell responses to *C. trachomatis*. Live *C. trachomatis* induced stronger IFN-γ–producing CD4^+^ T-cell responses than HK bacteria. A *C. trachomatis* OMP vaccine consisting of MOMP, PmpE, PmpG, and PmpH elicited only modest CD4^+^ T-cell responses, whereas the *C. muridarum*–based vaccine intrOv generated robust, cross-serovar immunity. The MIMIC system not only provides a promising tool for studying cellular responses to *C. trachomatis* infection in humans but also offers a practical, cost- and time-efficient platform for assessing various vaccine candidates in human settings, thereby accelerating the development of safe and effective vaccines for chlamydia and other infectious diseases.

## Supplementary Material

vkag080_Supplementary_Data

## Data Availability

The data generated or analyzed during this study are included in this published article and its online supplementary material. Original data are available upon reasonable request.
