# Supplementary material for: Immunological evaluation of vaccines to prevent Chlamydia trachomatis infection using the human in vitro MIMIC system
Source: J Immunol. 2026 Apr 21;215(4):vkag080. doi: 10.1093/jimmun/vkag080 (PMC13471025; doi:10.1093/jimmun/vkag080)
Supplement: Supplementary file 1 [file vkag080_Supplementary_Data.pdf]

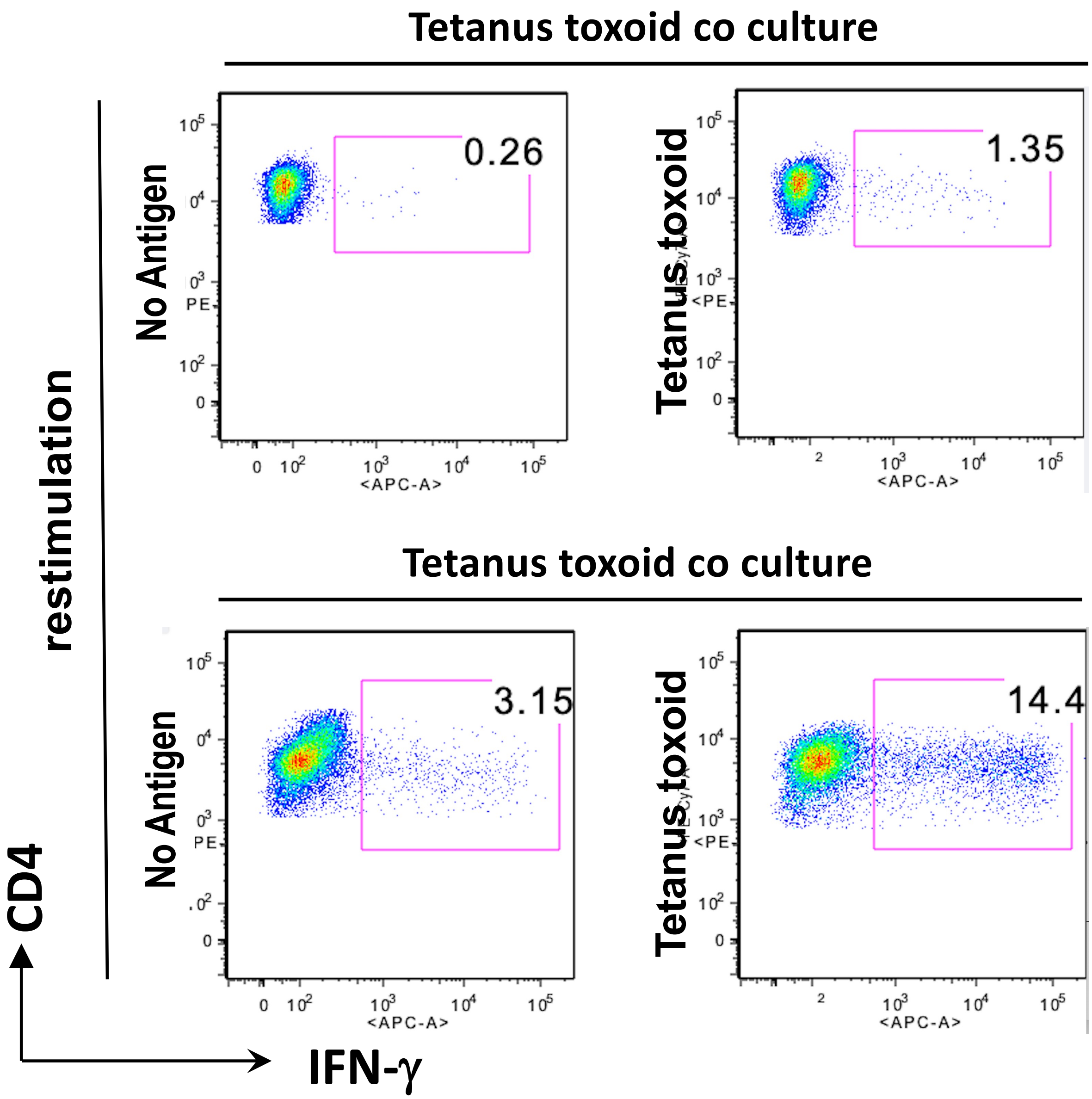

Supplemental Figure 1: DC/CD4 T cell coculture induced human CD4 T cell responses against tetanus toxoid. Tetanus toxoid was used as a positive antigen to establish the approach of DC/CD4 co-culture system. Monocyte-derived DCs were pulsed with tetanus toxoid and cocultured in serum-free X-VIVO15 medium with autologous CD4 T cells for 14 days. Then the cells were harvested and evaluated for IFN-γ expression by flow cytometry following a 7-h restimulation with tetanus toxoid or no antigen-pulsed DCs. The top row was from a subject vaccinated with tetanus toxoid four years earlier. The bottom row was from a subject vaccinated with tetanus toxoid three weeks earlier.

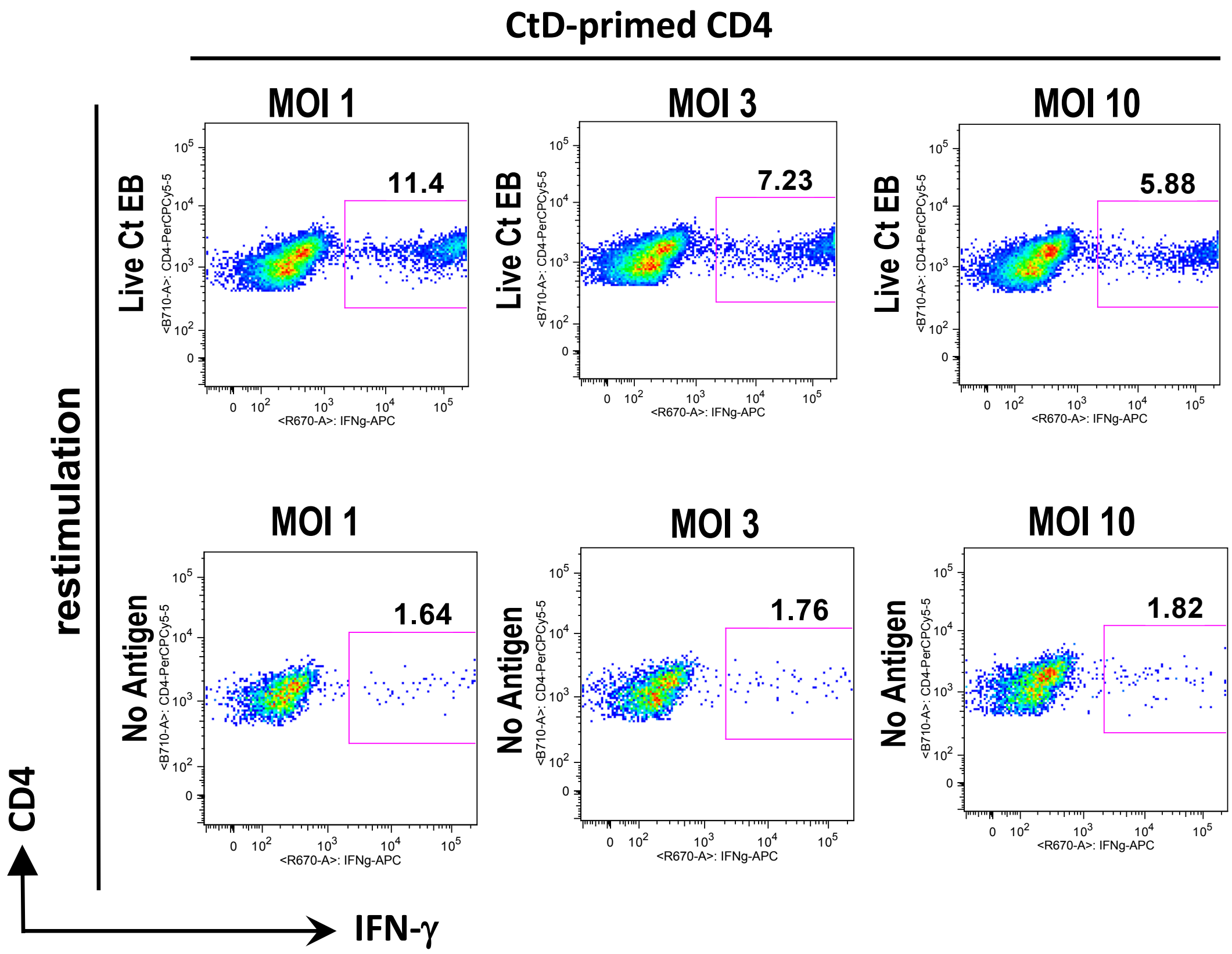

Supplemental Figure 2. *C. trachomatis* titration for dendritic cell infection in the MIMIC system. Monocyte-derived DCs from a *C. trachomatis*-naïve subject were pulsed with EBs at MOI 1, 3, or 10 and cocultured with autologous $CD4^+$ T cells in serum-free X-VIVO15 medium for 14 days. Cells were then harvested and assessed for IFN-γ expression by flow cytometry after a 7-h restimulation with *C. trachomatis* EBs or unpulsed DCs. DCs infected at MOI 1 induced the highest frequency of IFN-γ–producing $CD4^+$ T cells and were used in subsequent MIMIC assays.

Dynamics of cytokines in Ct primary CD4 responses

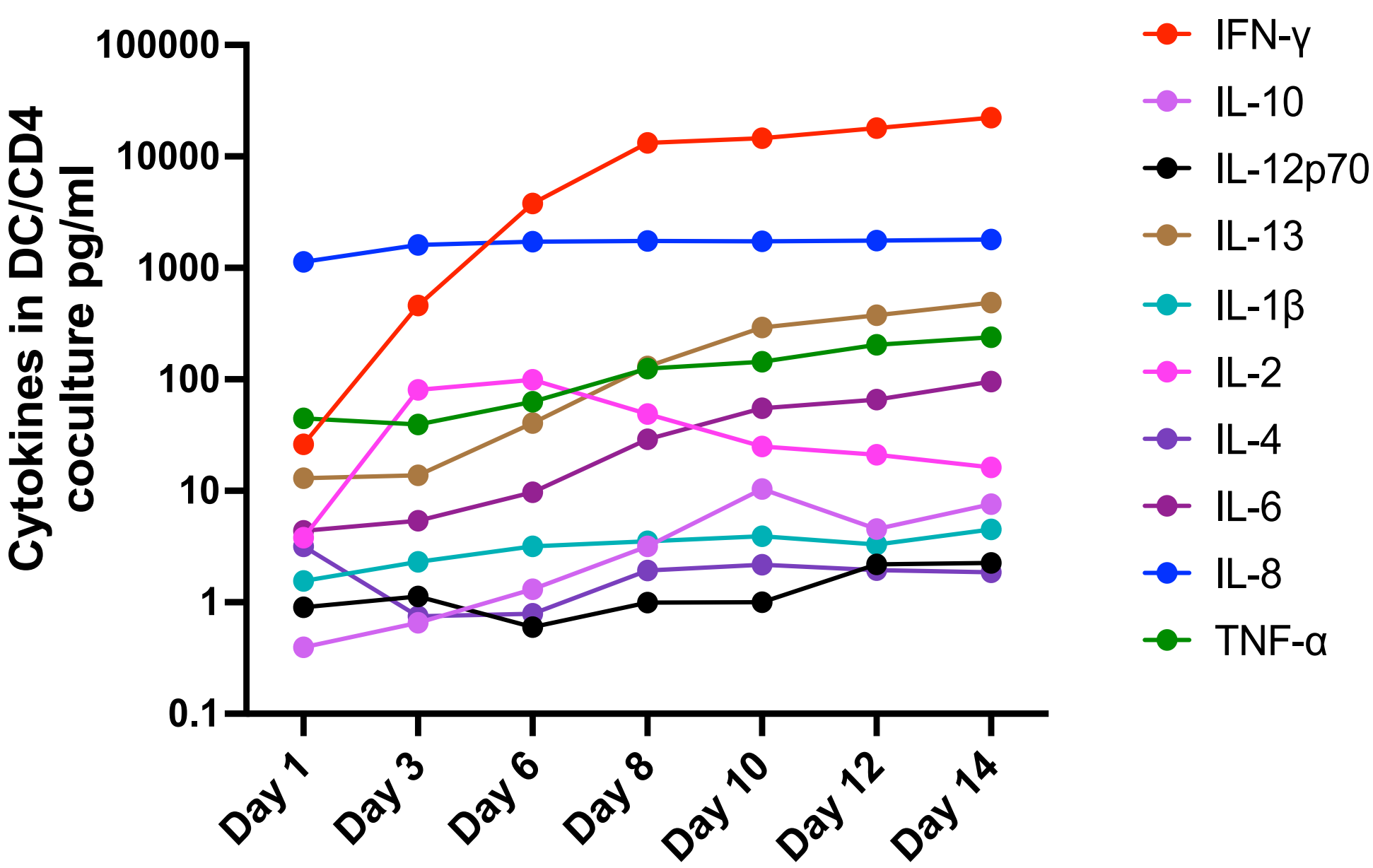

Supplemental Figure 3. Dynamics of cytokines in primary CD4$^+$ T cell responses to C. trachomatis. Monocyte-derived DCs from a *C. trachomatis* naïve subject were pulsed with live C. trachomatis EBs and cocultured with autologous CD4$^+$ T cells in serum-free X-VIVO15 medium for 14 days. Supernatants were collected at the indicated time points, and 10 cytokines were measured by MSD assay from a C. trachomatis-naïve subjects.

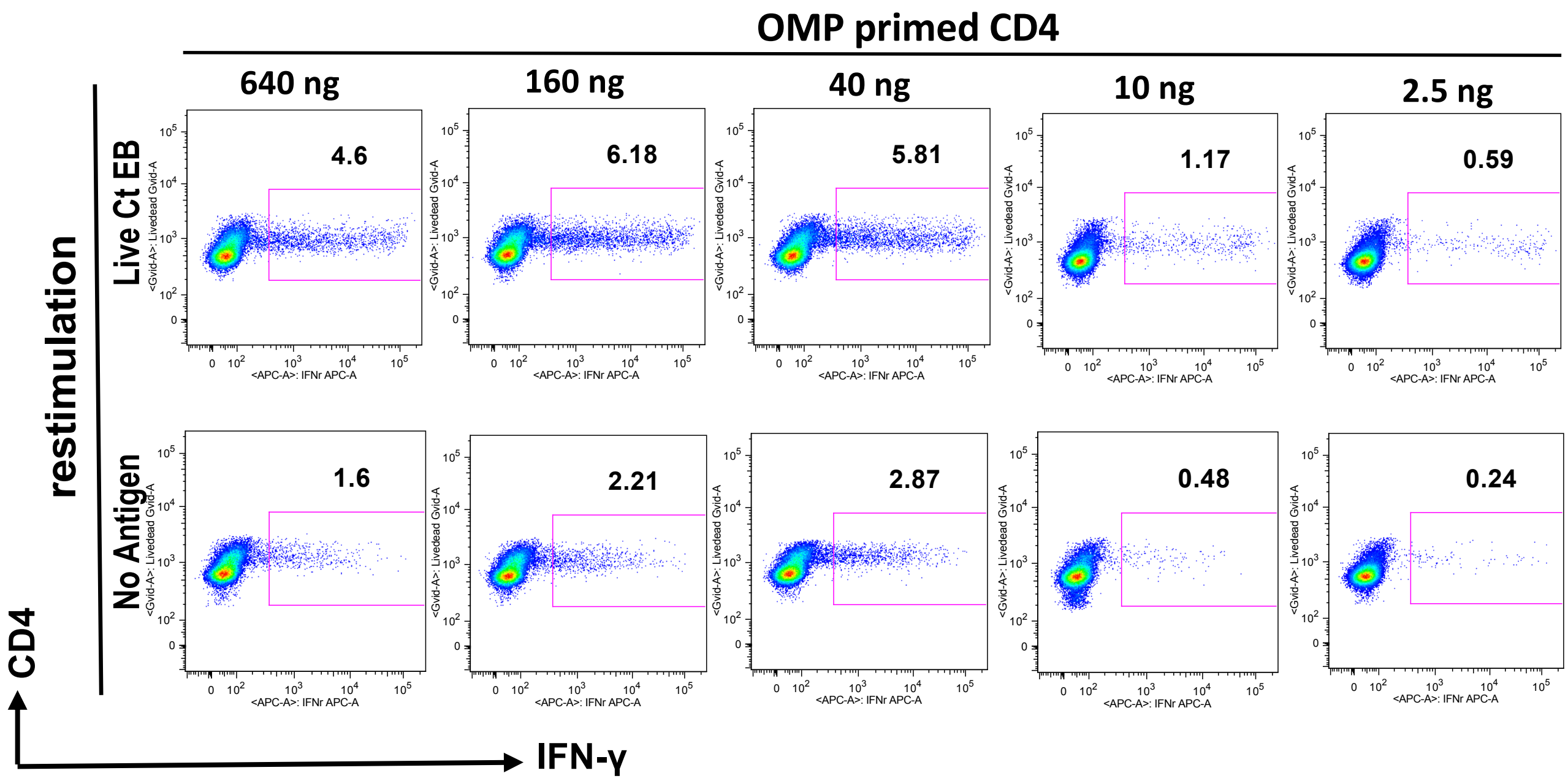

Supplemental Figure 4. OMP vaccine titration for dendritic cell pulsing in the MIMIC system. Monocyte-derived DCs from a *C. trachomatis*-naïve subject were pulsed with the OMP vaccine at total protein concentrations of 640, 160, 40, 10, or 2.5 ng/ml and cocultured with autologous $CD4^+$ T cells in serum-free X-VIVO15 medium for 14 days. Cells were harvested and analyzed for IFN-γ expression by flow cytometry after a 7-h restimulation with *C. trachomatis* EBs or unpulsed DCs. DCs pulsed with 160 ng/ml OMP vaccine (40 ng/ml each of PmpE, PmpG, PmpH, and MOMP) induced the highest frequency of IFN-γ–producing $CD4^+$ T cells and were used in subsequent MIMIC assays.

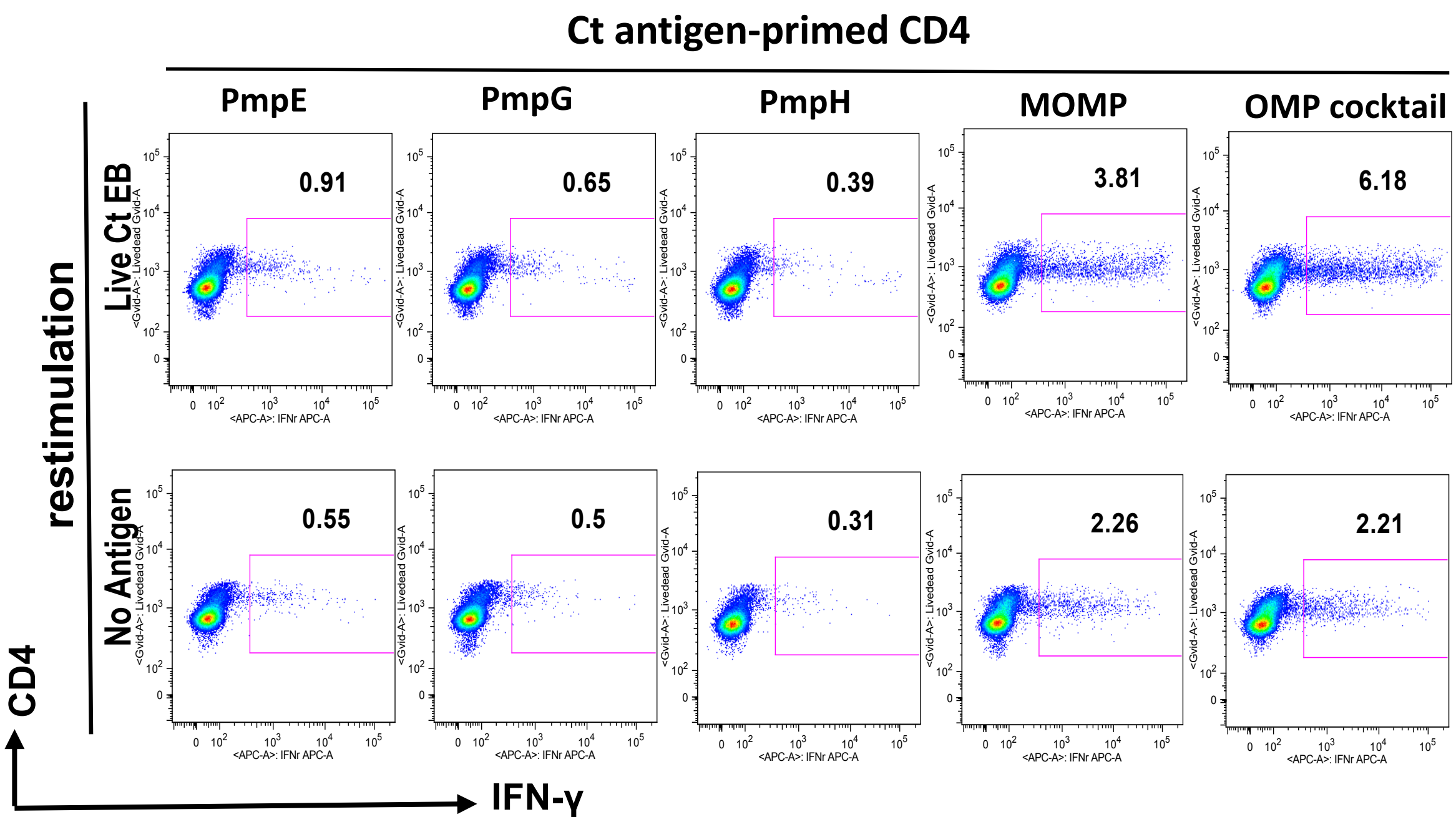

Supplemental Figure 5. Frequency of *C. trachomatis*-specific IFN-γ-producing CD4⁺ T cells induced by individual OMP antigens in the MIMIC system. Monocyte-derived DCs from a *C. trachomatis*-naïve subject were pulsed with PmpE (40 ng/ml), PmpG (40 ng/ml), PmpH (40 ng/ml), MOMP (40 ng/ml), or the OMP vaccine (160 ng/ml; 40 ng/ml each of PmpE, PmpG, PmpH, and MOMP) and cocultured with autologous $CD4^+$ T cells in serum-free X-VIVO15 medium for 14 days. Cells were harvested and analyzed for IFN-γ expression by flow cytometry after a 7-h restimulation with *C. trachomatis* EBs or unpulsed DCs. MOMP elicited the strongest IFN-γ response, followed by PmpE, PmpG, and PmpH. The OMP vaccine generated a cumulative effect, inducing stronger *C. trachomatis*–specific IFN-γ responses than any individual antigen.

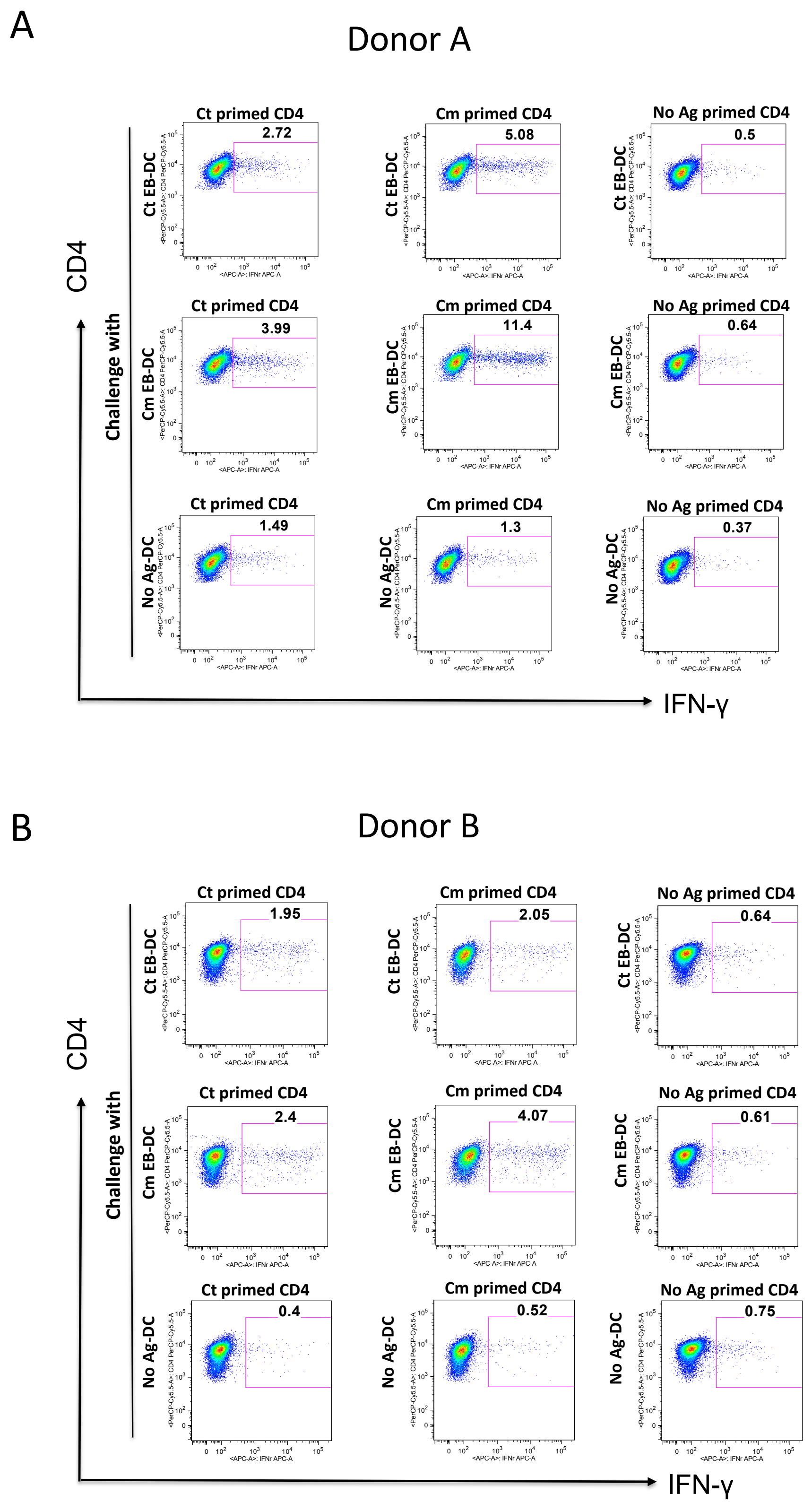

Supplemental Figure 6. *C. muridarum* induced potent human CD4 T cell responses against the *C. trachomatis* challenge. Cm-primed CD4 T cells from two CT naïve subjects produced potent IFN-γ responses against the Ct challenge in the MIMIC system.

Donor A: Titration of *Chlamydia muridarum* wild type (Cm_wt) to induce human *C. trachomatis*- specific IFN-γ-producing $CD4^+$ T cells (%)			
	Cm_wt primed CD4		
	MOI 10	MOI 3	MOI 1
Ct EB-DC sti	4.56	4.63	2.7
No Ag-DC sti	1.97	1.99	1.95
Specific Res	2.59	2.64	0.75

Donor B: Titration of Cm_wt and Cm_IntrOv to induce human *C. trachomatis* - specific IFN-γ-producing $CD4^+$ T cells (%)						
	Cm_intrOv-primed CD4			Cm_wt -primed CD4		
	MOI 10	MOI 3	MOI 1	MOI 10	MOI 3	MOI 1
Ct EB-DC sti	11.7	15.9	15.1	8.88	13.2	11.5
No Ag-DC sti	4.65	5.94	7.97	5.53	4.41	6.54
Specific Res	7.05	9.96	7.13	3.35	8.79	4.96

Donor C: Titration of Cm_wt and Cm_IntrOv to induce human *C. trachomatis*- specific IFN-γ-producing $CD4^+$ T cells (%)					
	Cm_intrOv primed CD4		Cm_wt primed CD4		no Ag-primed CD4
	MOI 10	MOI 3	MOI 10	MOI 3	
Ct EB-DC sti	3.53	5.02	3.73	5.96	1.72
No Ag-DC sti	2.9	3.64	2.69	4.33	2.41
Specific Res	0.63	1.38	1.04	1.63	-0.69

Supplemental Figure 7. Titration of *Chlamydia muridarum* strains to induce human *C. trachomatis*-specific IFN-γ-producing $CD4^+$ T cells. Monocyte-derived DCs from three *C. trachomatis*-naïve donors were pulsed with Cm_wt or Cm_IntrOv at varying doses and cocultured with autologous $CD4^+$ T cells in serum-free X-VIVO15 medium for 14 days. IFN-γ expression was measured by flow cytometry after a 7-h restimulation with *C. trachomatis* EBs or unpulsed DCs, and results are presented as specific responses for Donors A–C. An MOI of 3 was chosen for both Cm_wt and Cm_IntrOv for DC pulsing in subsequent MIMIC assays.

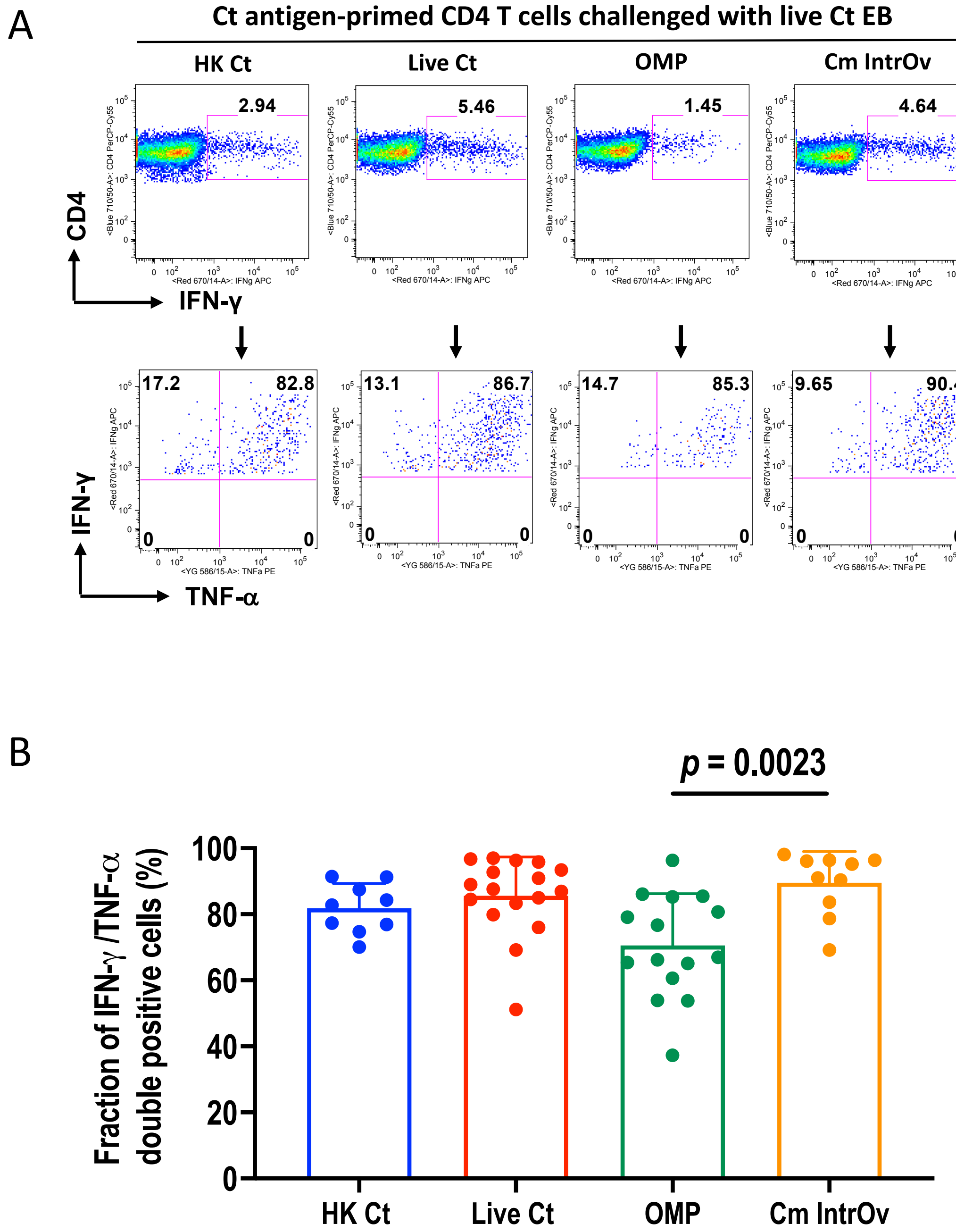

Supplemental Figure 8. Analysis of IFN-γ/TNF-α double-positive cells among total *C. trachomatis*-specific IFN-γ-producing CD4⁺ T cells in the MIMIC system. CD4⁺ T cells primed with heat-killed *C. trachomatis* (HK Ct), live *C. trachomatis* (Live Ct), OMP vaccine, or Cm IntrOv vaccine were challenged with live Ct EB-pulsed DCs, and IFN-γ/TNF-α double-positive cells were assessed by flow cytometry. **(A)** Representative flow cytometry plots showing *C. trachomatis*-specific IFN-γ-producing CD4⁺ T cells (upper panels) and IFN-γ/TNF-α double-positive cells (lower panels) induced by HK Ct, Live Ct, OMP, or Cm IntrOv in a *C. trachomatis* naïve subject. **(B)** Fraction of IFN-γ/TNF-α double-positive cells within the total IFN-γ-producing CD4⁺ T-cell population in the HK Ct, Live Ct, OMP, and Cm IntrOv groups. Data are presented as mean ± SD. Two-tailed unpaired t test was used for statistical analysis. p value of less than 0.05 was considered statistically significant.
